# A systematic review of interventions to promote HPV vaccination globally

**DOI:** 10.1186/s12889-023-15876-5

**Published:** 2023-06-29

**Authors:** Cam Escoffery, Courtney Petagna, Christine Agnone, Stephen Perez, Lindsay B. Saber, Grace Ryan, Meena Dhir, Swathi Sekar, Katherine A. Yeager, Caitlin B. Biddell, Purnima Madhivanan, Stephanie Lee, Amanda S. English, Lara Savas, Eliza Daly, Thuy Vu, Maria E. Fernandez

**Affiliations:** 1 Rollins School of Public Health, Emory University, 1518 Clifton Road, NE, Atlanta, GA 30322, 404-727-4701 USA; 2 Department of Population and Quantitative Health Sciences, University of Massachusetts Chan Medical School, Worcester, MA USA; 3 Nell Hodgson Woodruff School of Nursing, Emory University, Atlanta, GA USA; 4 Gillings School of Global Public Health, University of North Carolina at Chapel Hill, Chapel Hill, Durham, NC USA; 5 Mel & Enid Zuckerman College of Public Health, University of Arizona, Tucson, USA; 6 Institute for Health Disparities, University of North Texas Health Science Center, Fort Worth, TX USA; 7 Center for Health Promotion and Prevention Research, School of Public Health, University of Texas Health Science Center at Houston, Houston, TX USA; 8 Prevention Research Center, College of Public Health, University of Iowa, Iowa City, IA USA; 9 Health Promotion Research Center, School of Public Health, University of Washington, Seattle, WA USA

**Keywords:** Adolescent, Young adults, Health promotion, HPV vaccination, Vaccination, Interventions

## Abstract

**Background:**

Despite the human papillomavirus (HPV) vaccine being a safe, effective cancer prevention method, its uptake is suboptimal in the United States (U.S.). Previous research has found a variety of intervention strategies (environmental and behavioral) to increase its uptake. The purpose of the study is to systematically review the literature on interventions that promote HPV vaccination from 2015 to 2020.

**Methods:**

We updated a systematic review of interventions to promote HPV vaccine uptake globally. We ran keyword searches in six bibliographic databases. Target audience, design, level of intervention, components and outcomes were abstracted from the full-text articles in Excel databases.

**Results:**

Of the 79 articles, most were conducted in the U.S. (72.2%) and in clinical (40.5%) or school settings (32.9%), and were directed at a single level (76.3%) of the socio-ecological model. Related to the intervention type, most were informational (*n* = 25, 31.6%) or patient-targeted decision support (*n* = 23, 29.1%). About 24% were multi-level interventions, with 16 (88.9%) combining two levels. Twenty-seven (33.8%) reported using theory in intervention development. Of those reporting HPV vaccine outcomes, post-intervention vaccine initiation ranged from 5% to 99.2%, while series completion ranged from 6.8% to 93.0%. Facilitators to implementation were the use of patient navigators and user-friendly resources, while barriers included costs, time to implement and difficulties of integrating interventions into the organizational workflow.

**Conclusions:**

There is a strong need to expand the implementation of HPV-vaccine promotion interventions beyond education alone and at a single level of intervention. Development and evaluation of effective strategies and multi-level interventions may increase the uptake of the HPV vaccine among adolescents and young adults.

**Supplementary Information:**

The online version contains supplementary material available at 10.1186/s12889-023-15876-5.

## Background

The human papillomavirus (HPV) is the most common infection that can lead to cancer later in life. There are 570,000 incident cancer cases per year in females and 60,000 incident cancer cases in males attributable to HPV globally [1].HPV can lead to cancers of the cervix, vagina, and vulva for females, penis cancer for males, and anus and oropharyngeal cancers for both [1]. The World Health Organization has a vision to eliminate HPV-related cancers, particularly cervical cancer, worldwide by 2030 [2]. Similarly, in the U.S., Healthy People 2030 has an objective to increase the proportion of adolescents who receive recommended doses of the HPV vaccine from a baseline of 48.0% to 80.0% [3].

HPV vaccination can prevent more than 90% of cancers due to HPV infections [4, 5]. Vaccination starts at age 9 and the catch up is recommended through age 26. If not adequately vaccinated, persons up to the age of 45 can be considered for vaccination but with shared decision-making between the patient and provider [6]. Primary prevention is from ages 9–14 globally [7]. The HPV vaccine is commonly recommended during routine vaccinations to children ages 11–12 and there is a push from public health professionals and providers to start as early as 9 in the U.S [8]. Globally, an estimated 15% of girls are fully vaccinated against HPV [9]. In the U.S., about 58.5% of adolescents were up-to-date on HPV vaccination in 2020, with 61% of females being fully vaccinated versus 56% of males [10]. Public health efforts are needed to increase the global rates of HPV vaccination.

Worldwide, there have been a few reviews of interventions focused on improving HPV vaccination rates [11–15]. Interventions to promote HPV vaccination have typically targeted parents, adolescents, young adults, and providers.. HPV vaccination interventions have targeted various socio-ecological levels that influence HPV vaccination to ultimately effect change. Some focus only on the individual level (e.g., via education such as informational text included with reminders), whereas others may include changes to policy (e.g., via formalized requirements, such as school mandates). Multi-level and multi-component interventions are increasingly used [12, 13, 15] and address health disparities [16, 17]. Multi-level interventions target two or more levels of influence at or around the same time; the approaches implemented at each level typically may vary in type (e.g., behavioral, health systems, or policy) [16, 18]. It is important to understand the wide range of levels that can be utilized in interventions from single-level to multi-level and how those levels can impact the desired outcome of vaccination.

This study aimed to conduct a systematic review of HPV interventions by synthesizing literature published from May 2015 to March 2020, related to promoting HPV vaccine uptake and/or completion in the U.S and internationally. A previous systematic review and meta-analysis in the United States found a combination of provider- and community-level interventions were effective [11]. Our review was intended to update this review of interventions for HPV vaccine promotion with more rigorous methodology, including exploration of sources of heterogeneity and quality assessment. Another purpose of the study was to improve the understanding of multi-level interventions for HPV vaccine promotion. The review questions included: 1) What are the targeted audiences and levels of intervention for HPV vaccination interventions?, 2) What are common components of the interventions?, 3) What were facilitators and barriers to implementation of the vaccination interventions?, and 4) What are the study outcomes measured including the rates of HPV vaccination initiation and completion and their effectiveness? Our resulting study provides a strong contribution to the literature that can be used to inform future promotion efforts that aim to increase HPV vaccine uptake.

## Methods

We conducted a systematic review of the peer-reviewed published literature, using methods following the PRISMA guidelines [19]. The team included cancer control researchers and master’s and doctoral students in public health and nursing fields.

### Search strategy

The lead author, in collaboration with a health sciences librarian, created a search strategy using text and MeSH terms (Supplemental Table 1). We searched for relevant articles in six bibliographic databases, including Medline, CINAHL, Embase, Web of Science, Cochrane Reviews, and SCOPUS. Some of the keywords searched alone or in combination were children, pediatric, young adult, parent, behavioral therapy, prevention, and human papilloma virus. An additional manual search was performed of the bibliographies of relevant studies identified from the database search. The team reviewed the articles found in the search and removed duplicates.

### Inclusion criteria

To be included in the review, an article had to: a) aim to increase HPV vaccination through at least one intervention; b) report an outcome based on the intervention (e.g., increase knowledge of HPV, report on HPV vaccine outcomes determined either by self-report or medical records; c) be published between May 2015 through March 2020; and d) be published in English. Studies that tested single or multi-level interventions were included. Screening was conducted in two stages with the initial stage evaluating titles and abstracts reviewed by 3 authors (CE, CA, and MD), and a second stage screening full text articles independently reviewed by the same 3 authors. Discrepancies were resolved through discussion at team meetings. Studies were excluded if they did not describe a primary intervention aimed at increasing HPV vaccination, were systematic reviews or articles with just a program description, or had no study outcomes. Those that met eligibility through abstract review were included in the full-text review. After the full article review, the articles were examined further to see if they met the eligibility criteria, and 33 were excluded.

### Data extraction

We retrieved the full text of eligible studies for review and abstraction. We then created a detailed codebook for data collection. Data extraction tables for the article and quality assessment were developed and maintained in an Excel database. They were modified following discussions between three reviewers before data extraction. Data extracted included study location, target population, sample description, and setting; intervention details consisted of study design, description of the intervention (e.g., control group components, if applicable), level(s) of intervention, delivery and barriers to implementation and vaccination, and outcomes of the study. We piloted the forms with five studies and made refinements to the codebook and Excel database. We invited cancer and implementation science researchers from the Cancer Prevention and Control Research Network [20] and doctoral and MPH students from the participating institutions to be trained as data abstractors and abstract data from the final included articles. There were a total of 15 reviewers (CA, CP, CE, MD, SS, CB, MF, AE, LS, ED, GR, KY, SL, TV, and PM). For quality control, we had 2 abstractors for each study, and we merged the data when consensus was reached for each article. The abstractors also performed study quality assessment for the articles they abstracted. The pair of abstractors came to an agreement if there were discrepancies. If there was a disagreement or question about a study quality answer, then the core team (CA, CP, and CE) had a discussion and came to an agreement on the study quality question.

### Quality assessment

For this assessment, we employed the NCI Quality Rating assessment for Pre and Posttest Designs to conduct quality assessment of the included articles [21]. This assessment included 12 items which included: whether the objectives, intervention, and eligibility requirements were clearly stated, had a sample adequate for confidence in the data, had a loss to follow-up of 20% or less, and measured changes in outcomes of interest before and after the intervention.

### Synthesis of the results

We compiled all article abstractions into one database. We ran descriptive statistics and created summary scores for study setting and program component descriptions. The Community Guide categories (education, technology, vaccine access, incentive, provider education, health system change, community wide campaign, and policy) were used to organize the interventions into informational; behavioral change for participants, providers or both; or environmental (small-no government involvement such as organizational policy change or large policy-formal laws, rules or regulations, national or local government involvement). These categories also were applied in the Walling et al. systematic review [12]. We also created summary tables for study characteristics, outcomes, and quality ratings. The primary outcome was HPV vaccine initiation and/or completion, although we reported on other outcomes related to HPV vaccination determinants, or factors to increase vaccination (i.e., parental knowledge, awareness, self-efficacy, acceptability, attitudes and beliefs, and vaccine intention). We examined the range of HPV vaccine initiation and completion for adolescents and/or young adults.

## Results

**Fig. 1:**
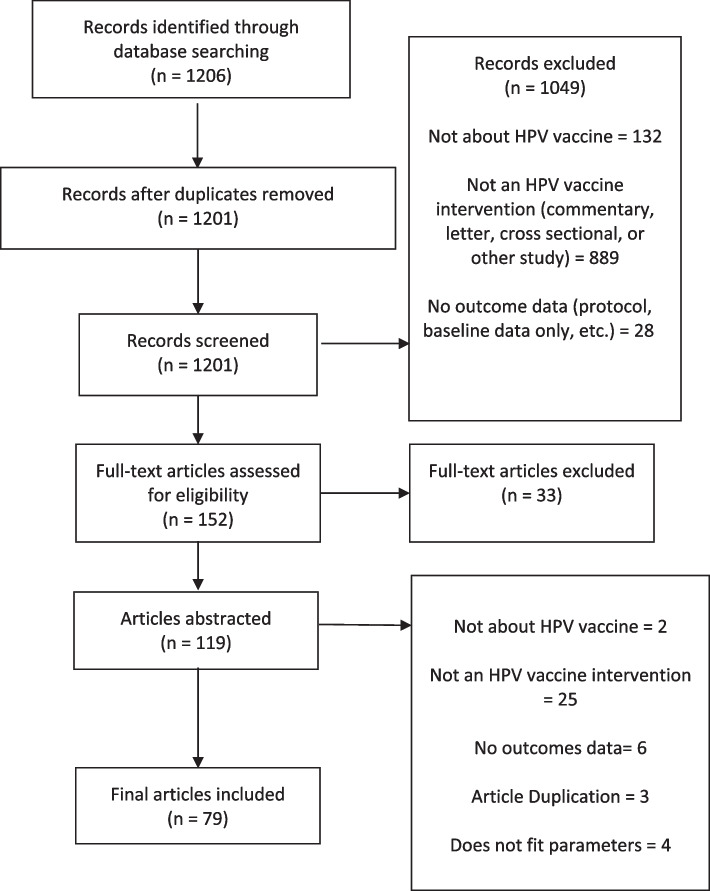
HPV Vaccination intervention systematic review flowchart

The search identified 1,201 studies after removing duplicates. As a result of the title and abstract screen, 1,045 studies were excluded due to not being an intervention study or not reporting outcomes. The full-text of the remaining 152 articles were reviewed, leading to the exclusion of an additional 72 articles that did not have descriptions of the intervention or outcome data. This resulted in 79 articles included in the review for data extraction (Fig. 1). Table 1 shows the main characteristics of the included studies published between 2015 and 2020.

**Table 1:** Summary characteristics of included studies

				Level(s)												
**Author, Year**	**Location**	**Study Design**	**Level: single or multi**	**Ind**	**Int er**	**Prov**	**Org**	**Comm**	**Po** **l**	**Setting:**	**Study Audience(s)** **Characteristics** **(Sample, Race %, Gender, Ages)** NR = Not reported	**Intervention Description**	**Measures** **Individual** **Parent** **Provider** **System** **Community** **Policy**	**Vaccination Outcomes** **Initiation (VI) or Series Completion (VC)** NC: Not collected NR: Not Reported	**Other Outcomes**	**Implementa-tion Facilitators (F) or Barriers (B)** NR: Not Reported
**Austin et al., 2019 ** [22]	Jacksonville, FL, USA	Quasi-Experiment	Single			X				Family practices affiliated with large hospital system	Sample: preteens (11–12 years old), adolescents (13–17 years old), and young adults (18–26 years old) Race: NR Gender: NR Age: *Preteens:* 11–12, *Adolescents:* 13–17, *Young Adults:* 18–26	An education session, resource packet, e-mail links, and site visits with performance feedback; a resource packet focused on improving HPV vaccine uptake in the family practice setting CG: Beh Interv Providers	Healthcare Providers	VI: initiation rates increased 2.7% for combined sexes (2.5% for females, 2.7% for males) Female 11- to -12-year age group showed a 4.8% increase in vaccine initiation; male 13- to 17-year age group showed a 3.8% increase in vaccine initiation VC: rates showed a minimal increase of a 0.2% to 0.6% increase across both sexes with the 0.6% increase in the female 18- to 26-year age group	Knowledge: 12% improvement in HPV knowledge after the educational intervention (81.3% vs 93.1%)	F: NR B: NR
**Baxter & Baraita, 2011 ** [23]	University of Guelph, Ontario, Canada	Experiment	Single	X						Students at the undergraduate school- university of Guelph	Sample: 193 students Race: White 161 (83%), South Asian 9 (5%), South East Asian 5 (3%), Black 5 (3%), West Asian 1 (0.5%), Latin American 1 (0.5%), Arab 1 (0.5%), and Other 10 (5%) Gender: 100% Female Age: 17–23, M = 18.43, (SD = 0.93)	• An educational HPV message that emphasized the benefit of the HPV vaccine for sexually inexperienced women • Participants were provided with one out of three types of messages regarding the HPV vaccine: 1) minimal information that avoided all mention of sexual transmission; 2) detailed information about sexual transmission; and 3) tailored information for sexually inexperienced women CG: Information	Young Adults	NC	Knowledge: those in tailored information conditions knew significantly more about HPV and the vaccine than those in the control condition, no significant difference Intention to get vaccine: significant difference across conditions for women with no sexual experience	F: NR B: NR
**Bennett et al., 2015 ** [24]	Ann Arbor, Michigan, USA	Experiment	Single	X						Midwestern university	Sample: NR Race: Hispanic 30, Asian 98, Non-Hispanic Black 36, Non-Hispanic White 445, and Other/multiracial 52 Gender: 100% Female Age: 18–26	• MeFirst intervention website was a unique, tailored website automatically configured for the individual participant based on their baseline survey responses • The topic pages had factual information on HPV and the HPV vaccine, including statistics on the incidence of HPV infection and cervical cancer, risks associated with HPV infection, costs of vaccination, safety and efficacy of the HPV vaccine, and suggestions for how to talk to a doctor about the vaccine CG: Beh Interv Patient Decision Support	Young Adults	VI: 8% over a 3-month period; no difference in effect of individually tailored and nontailored educational materials on vaccine uptake rates	Knowledge: Knowledge of the HPV vaccine increased from baseline (32% to 50%)	F: NR B: Practical barriers- cost
**Berenson et al., 2015 ** [25]	Galveston, TX, USA	Quasi-Experiment	Single		X					Healthcare clinics	Sample: 427 participants Race: White 199 (67.0%), African American 40 (9.4%), Hispanic 69 (16.2%), Asian American 73 (17.1%), Other 26 (6.1%), Unknown 20 (4.7%) Gender: Female 67%, Male 31.6%; Unknown 1.6% Age: 74.2% < 30 yrs, 14.5% 30–49, 9.6% 50 + , 1.6% unknown	30 min presentation on HPV and the HPV vaccine CG: Behav Interv Providers	Healthcare Providers	NC	Knowledge: On average, knowledge scores significantly improved from 8 to 15 after the presentation (maximum possible score 16) (*P* < .001), irrespective of specialty, race/ethnicity, gender, and age	F: NR B: NR
**Berenson et al., 2016 ** [26]	Galveston County, TX, USA	Non- Experiment	Single	X						University of Texas Medical Branch (UTMB) at five prenatal clinics in Galveston County, Texas	Sample: 500 female young adults Race: Non-Hispanic White 141 (24.5%), Non-Hispanic Black 120 (20.9%), Hispanic 307 (53.4%), and Non-Hispanic others 7 (1.2%) Gender: 100% Female Age: 16–26	• Counseling for pregnant and postpartum women about HPV and the HPV vaccine • Eligible patients were then offered written materials and personal counseling about HPV and the vaccine CG: Information	Young Adults	VI: increase from 25.4% before to 80.8% VC: 15.5% to 65.1% for the entire study population; Those who were Hispanic (2.33 OR for VI, 2.08 OR for VC), or had received an influenza vaccination in the last year were more likely to initiate and complete the HPV series in the program	NC	F: Patient Navigators B: Patients’ moving away/ changing phone number/ have more than two children
**Berenson et al., 2019 ** [27]	Galveston, TX, USA	Non- Experiment	Multi		X		X			Pediatric clinics	Sample: 2,162 Race: Hispanic 743 (34.4%), Black 632 (29.2%), White 737 (34.1%), and Asian/other 50 (2.3%) Gender: Female 47.2%, Male 52.8% Age: 9–10 yrs: 208 (9.6%), 11–12 yrs: 971 (44.9%), 13–14 yrs: 536 (24.8%), 15–17 yrs: 447 (20.7%)	• 45 min lectures to faculty, residents, medical students, and staff working in the clinics • Parents of unvaccinated or incompletely vaccinated children were informed about HPV vaccination while in private clinic rooms; parents were offered personal counseling and given CDC handouts in English or Spanish CG: Pat/Providers Targeted Inter	Healthcare Providers	VI: 66.9% of eligible patients received the first dose VC: Of those that began between 2/1/15 and 8/31/16, 864/930 (93.0%) completed the series by 8/31/2017	NR	F: Sending out multiple reminders, and calling parents the day before the appointment as a reminder B: Assuring vaccine safety among parents and cost-intensive by using patient navigators
**Bonafide & Vanable, 2015 ** [28]	Boston, MA- Northeastern University, USA	Experiment	Single	X						Northeastern University	Sample: 200 underguate students Race: White 69%, Asian or Pacific Islander 17%, African American 11%, and Latino 4% Gender: 100% Male Age: 19 (SD = 2.2)	Computer-administered surveys and informational interventions, varied in inclusion or exclusion of altruistic motives level of emphasis on male-specific HPV-related illnesses and vaccine benefits CG: Information	Young Adults	NC	Acceptability: significant differences in vaccine acceptance based on intervention condition	F: NR B: NR
**Botha et al.**, **2015 ** [29]	Western Cape, and Gauteng province, South Africa	Non- Experiment	Single	X						Primary Schools	Sample: 2046 girls Race: NR Gender: 100% Female Age: 9 and older—grades 4, 5, 6, 7	Information on cervical cancer and HPV vaccination was provided to 19 primary schools in Western Cape and Gauteng provinces; girls with parental consent and child assent were vaccinated during school hours at their schools CG: Environ Small Policy	Children	VI: 2,030 girls (99.2% of the consented) VC: 1,782 (87.8%) girls received all three doses	NC	F: Verbal, interactive information sessions when literacy levels are low B: NR
**Calo et al., 2019 ** [30]	Illinois, Michigan, and Washington, USA	Quasi-Experiment	Single				X			Primary Care Clinics	Sample: 193-nurses (31%–46%) or clinic managers (18%–32%); physicians (5%–10%) of survey respondent Race: NR Gender: NR Age: NR	In-person or webinar HPV Assessment, Feedback, and eX-change (AFIX**) quality improvement** (QI) coaching session which consisted of: • A single session, designed to be ~ 60 min both in person and online • QI coaches meeting with providers to discuss the clinic’s immunization coverage levels with continue medical education (CME) • Sharing the clinic’s immunization coverage assessment CG: Beh Interv Providers	Health Systems	NC	Acceptability: At 6-month follow-up, 54% somewhat or strongly agreed that implementing the QI activities improved their clinic’s HPV vaccine coverage levels	F: QI and coaching, convenience, helpfulness, facilitation, acceptability, delivery costs B: Limited staff time, time constraints during patient visits, staff turnover, lack of support from leaders, limited stock of HPV vaccine
**Carolan et al., 2018 ** [31]	Northwestern England	Experiment	Single	X						Secondary School	Sample: Students aged 14–15:63 Race: Asian/Asian British 3 (5.45%), White British 59 (93.65%), Asian/Asian British 3 (5.45%), and Mixed Ethnic Background 1 (1.59%) Gender: Female 46.03%, Male 53.97% Age: 11–15	In a two- group experimental study: • Group A received the digital game-based resource (*n* = 26) • Group B received a traditional PowerPoint lesson (*n* = 21) • Group C is the control group (*n* = 16) Both groups A and B then participated in one short session education and worksheet CG: Information	Young Adults, Adolescents	NC	Knowledge: significant difference between the three groups for “I know all I need to know about vaccination and how it works”	F: NR B: NR
**Chigbu et al., 2017 ** [32]	Southeast Nigeria	Non- Experiment	Multi	X	X					Communities in Southeast Nigeria	Sample: 1327 women Race: NR Gender: 100% Female Age: 30 years and older and girls 9–13 years old for the vaccination arm of the study	House-to-house cervical and breast cancer prevention education; 1–1 basis with materials, told about availability of cervical/ breast cancer prevention services at local health facilities CG: Beh Interv Patient Decision Support	Children/adolescents, older adults ages 30 and older	VI: 33.2% (71/214)	Screening: Forty-two (3.2%) women had undergone cervical cancer screening before the intervention and after the intervention, 897 (67.6%) Awareness: 846 (94.3%) were not aware of cervical cancer screening	F: Selected nurses trained as Community health educators on cancer prevention and as cervical and breast cancers service providers B: NR
**Cipriano et al., 2018 ** [33]	Southern New Jersey, USA	Non- Experiment	Single	X						Parents of adolescents who go to federally qualified health centers in southern New Jersey	Sample: 75 parents of adolescents Race: White 29 (38.7%), Black: 19 (25.3%), Hispanic 19 (25.3%), Asian 1 (1.3%), and Other: 7 (9.3%) Gender: Female 64%, Male 36% Age: 11–16	A short, self-directed, computer-based learning PowerPoint presentation adapted from the CDC CG: Beh Interv Patient Decision Support	NR	NC	Knowledge: significantly higher post-intervention scores (*t* = − 10.585, *p* < .001) Attitudes: Parental Attitudes Module and the HPV Knowledge Survey pretest showed a positive moderate relationship (*R*s = .552, *p* < .001)	F: NR B: NR
**Cory et al., 2019 ** [34]	Pennsylvania, USA	Experimental	Single	X						University of Pennsylvania-affiliated clinic for OBGYN care	Sample: 256 Race: Black 207 (80.6%), Asian 9 (3.5%), American Indian or Alaskan Native 3 (1.2%), Native Hawaiian or other Pacific Islander 3 (1.2%), White 13 (5.1%), and Other 21 (8.2%) Gender: 100% Female Age: 12–34	One of three study arms: • control (no educational intervention), • educational handout (one-page hand- out) • educational video (approximately eight- minute video) CG: Beh Interv Patient Decision Support	Adolescents/ Young Adults	VI: 41% of women initiated the series VC: 19% of women completed the series	Acceptability: Educational video: 51% of women were willing to accept vaccine; Educational handout: 33.3% Control: 28.2%	F: NR B: NR
**Darville et al., 2018 ** [35]	Southeastern USA	Experiment	Single	X						University	Sample: 6,000 men eligible for selection, 168 enrolled, 108 completed the protocol Race: White or Caucasian (*n* = 46; 42.6%), Asian (*n* = 35; 32.4%), Native Hawaiian or Pacific Islander (*n* = 16, 14.8%), Hispanic (*n* = 6; 5.6%), and Bi-racial or Multiracial (*n* = 5; 4.6%) Gender: 100% Male Age: 18–26	Use of avatar characters, which were assigned and customized, and perception of self (ideal vs. actual) on HPV risk perception, HPV vaccine self-efficacy and behavioral intent to receive the HPV Vaccine CG: Beh Interv Patient Decision Support	Young Adults	NC	Education: no statistical **significance** between avatar type and perception of self in the model for risk perception, self-efficacy, and behavioral intention	F: NR B: NR
**Davies et al., 2017 **[36]	Western and South Australia	Experiment	Single					X		40 high **s**chools	Sample: Intervention: (21 schools, 3806 students Control: (19 schools, 3159 students) Race: NR Gender: NR Age: NR	Schools in the intervention group were provided with study educational materials and were advised to use the materials before the first dose of the vaccine was administered; educational materials were in class activities, games, DVD, website, magazine CG: Beh Interv Patient Decision Support	Adolescents	NC	Knowledge: at 6 months, intervention schools 53% correct responses vs 32% control schools. Significant difference 20%, *p* = < 0.0001	F: Having a comprehensive user guide, supplement, and user-friendly, age-appropriate resources B: Time constraints
**Dawson et al., 2018 **[37]	Killeen, TX; Tacoma, WA; San Antonio, TX; Honolulu, HI; El Paso, TX; Colorado Springs, CO; San Diego, CA; Fort Riley, KS; Fort Wainwright, AK, USA	Non- Experiment	Single		X					Primary care clinics	Sample: 200 providers in 48 primary care clinics Race: NR Gender: NR Age: NR	Provider education at clinic level using a standardized, interactive 1 h educational session: the educational sessions (‘‘You are the Key to HPV Cancer Prevention” from the CDC); role-playing how to recommend and effectively communicate the importance of this vaccine to patients and parents CG: Beh Interv Providers	Health Systems	VI: significant difference between the number of the first dose of vaccines administered in July 2014, October 2014, and January 2015 (F(2,21) = 37.91, *p* < 0.001; first dose of the vaccine given was significantly higher compared to July 2014 (*p* = 0.001) and October 2014 (*p* = 0.002); No significant difference in the overall number of vaccines given at all clinics 6 months following the educational sessions [t(7) = 1.06, *p* = 0.324]	Education: interactive educational sessions in the Fort Hood Region led to significant increases in short-term retainment of the educational material on HPV vaccine information (pre- and post-test scores [t(f7) = -5.04, *p* < 0.001]	F: standardized, interactive educational sessions that stress strong provider recommendation, having champion encouraging QI projects B: mobile families, lost to follow up, lack of provider engagement, and incomplete vaccination records
**Dempsey et al., 2019 ** [38]	Central Colorado, USA	Experimental	Single	X						Singular health system (family medicine)	Sample: 1294 young adults enrolled Race: Hispanic 85.2%, White NH 12.6%, and Other NH 2.0% Gender: Female parents: 48.4%, Male parents: 51.6%; Young Adults: 100% Female Age: 9–17 (parent participants) or 18–26 (young participants)	A 3-armed randomized controlled trial: CHICOs (Combatting HPV Infection and Cancers, tailored intervention) to an untailored intervention -iPad-based version of the Vaccine Information Sheet from the Centers for Disease Control and Prevention (untailored intervention), or to usual care CG: Beh Interv Patient Decision Support	Children	VI: 265 adolescents, while only 18 young adults received an HPV vaccine dose during the study period VC: no significant differences in series completion among the CHICOS cohort/ intervention was compared to usual care (OR 1.6, 95% CI 0.8–3.2)	Intention: no differences between study arms in vaccination intention at baseline or post-intervention for either parents or young adults Acceptability: among young adults, no significant differences between 2 study arms in any of the vaccination uptake measures in the intention to treat analysis	F: NR B: NR
**Dempsey et al., 2018 ** [39]	Denver, Colorado, USA	Experiment	Multi			X	X			24 practices in Denver, CO	Sample: 188 medical professionals Race: White 54.9%, Black 4.5%, Other 7.9%, Missing 32.7% Ethnicity: Hispanic 12.4%, and NH 44.6% Gender: Female 50.3%, Male 49.7% Age: 11–17	Healthcare professional communication intervention with 5 components for multiple levels: 1. a fact sheet library that practices used to create practice specific fact sheets about HPV infection and vaccination, 2. a parent education website called “iVac” 3. a decision aid for HPV vaccination 4. communication training 5. “presumptive approach” Motivational Interviewing (MI) techniques CG: Beh Interv Providers	Adolescents	VI: Intervention: 42.9% vs Control 38.9% VC: Intervention: 72.4% vs Control 68.1%		F: NR B: NR
**DiClemente et al., 2015 ** [40]	Atlanta, GA, USA	Experiment	Single	X						Clinics which provide STI services	Sample: 216 participants Race: 100% Black/ African American Gender: 100% Female Age: 13–18	Girls OnGuard intervention condition: Viewed a 12-min interactive computer-delivered media presentation on HPV vaccination CG: Beh Interv Patient Decision Support	Adolescents	VI: 12% of participants (*n* = 24) received the first dose of HPV vaccine, with an equal number of participants in the intervention and comparison conditions VC: intervention group included more participants who completed the vaccine series (2 vs. 17 doses in the comparison group respectively; p = .12)	Susceptibility: 19.5% of respondents believed they were at risk of cervical cancer. 41.2% worried about getting cervical cancer Intervention: significant main effect of viewing information on willingness to vaccinate child F(1,684) = 7.992, p = .005, partial η2 = .012	F: NR B: Any administrative fees associated with vaccination
**Donahue et al., 2018 ** [41]	South, Midwest, West, and Northeast Regions in USA	Experiment	Single		X					National Web-based survey	Sample: 2,476 mothers Race: White 71.6%, African American 13.7%, Hispanic or Latino 12.7%, Asian 4.8%, American Indian 2.3%, Native Hawaiian/Pacific Islander 0.9%, and other 1.8% Gender: Female 56.7% Male 43.3% Age: 9–13	One of six health messaging interventions based on a 3 × 2 between-subjects factorial design (strength of recommendation x safety information) CG: Information	Parents	NC	Acceptability: main effect of safety information, F(1,684) = 7.99, *p* = .005, and perceived benefits of vaccination, F(1,684) = 221.64, *p* < .001) on mothers’ willingness to vaccinate	F: NR B: NR
**Dreyer, G et al., 2015 **[42]	Western Cape (WC) and Gauteng Province (GP), South Africa	Non- Experiment	Multi	X			X			Primary schools’ grades 4 -7	Sample: 906 women completed first questionnaire, 766 women completed second questionnaire Race: NR Gender: 100% Female Age: Parents M = 38, girls were in grades 4–7	Printed information was distributed to all girls: pamphlets invited parents to consent to vaccination of their daughters and to attend information events at schools CG: Information	Parents	NR	Knowledge: 30.8% (239/777) attained a knowledge score of 0/5 for cervical cancer and its symptoms, 9.1% remained score of 0 at posttest. After education, 62.9% had confirmed adequate knowledge of cancer screening, v. 30.6% before	F: NR B: NR
**Edwards & Hooper, 2019 ** [43]	Northeastern USA	Non- Experiment	Multi	X			X			School based health center	Sample: parents of adolescents in grades 9–12 who attended a school-based health center (SBHC) (36 students) Race: NR Gender: NR Age: NR	Quality Improvement Project: The Plan Do Study Act (PDSA) model was used as a baseline and was tailored to the intervention CG: Beh Interv Providers	Parent	NC	Acceptability: 15 consent forms (42%) were returned for HPV vaccine	F: NR B: limited face-to-face interactions with parents, students taking HPV consent home
**Esposito et al., 2018 ** [44]	Milan, Italy	Experiment	Single	X						NR	Sample: 917 unvaccinated adolescents were enrolled Control: 334 Education: 281 Website + education: 302 Race: NR Gender: NR Age: 11–18	Three study arms: To reduce risk of contamination, passwords for access to a website providing explanations through multiple choice questions on how the immune system works, details on vaccine-preventable diseases, and information on vaccines were given only to those randomized to arms 2 and 3; those in arm 3 also participated in a lecture on vaccines and vaccination regarding the same topics included in the internet presentation from medical experts in classrooms CG: Beh Interv Patient Decision Support	Adolescents	VI: no significant increase in vaccination coverage observed for the HPV vaccine (*p* = 0.27)	Other vaccines: significant increase in vaccination coverage was observed for TdaP and menACYW in the 2 groups using the website (*p* < 0.001)	F: NR B: face-to-face discussions regarding vaccines at school and at home
**Ford et al., 2020 ** [45]	South Carolina, USA	Non- Experiment	Single					X		Communities near intervention site	Sample: 276 adults Race: African-American or Black 255 (93.1%), White 17 (6.2%), and Other 2 (0.8%) Gender: Female 90.7%, Male 9.3% Age: < 50 27.4%, 51–64 34.8%, 65 + 37.8%	A cancer educational intervention: important cancer educational topics relevant to African American cancer mortality rates were highlighted CG: Beh Interv Patient Decision Support	Individual	NC	Knowledge: significant difference in pre/posttest knowledge on cervical cancer (*p* < 0.05)	F: community partners included leaders from the following organizations: churches, American Cancer Socity, cancer alliance B: NR
**Forster et al., 2017 ** [46]	London, UK	Experiment	Single				X			6 London Schools	Sample: year 8 girls in 6 schools Race: White 7.8%, African 3.6%, Other 4.9%, and Missing 83.8% Gender: 100% Female Age: 12–13	Two-arm cluster randomized feasibility trial Intervention: students had a chance to win a voucher if they returned a vaccination consent form CG: Beh Interv Patient Decision Support	Adolescents	NC	Acceptability: proportion of girls whose parents gave consent for vaccination was higher in the intervention arm (76%) than the standard invitation arm (61%)	F: NR B: NR
**Gerend, Murdock, & Grove, 2020 **[47]	Tallahassee, FL, USA	Quasi-Experiment	Multi	X			X	X		University	Sample: students and UHS Providers Race: NR Gender: Female 78%, Male 22% Age: NR	Two primary components: Student-directed campaign materials and provider directed training with encouragement to recommend HPV vaccine to all eligible students CG: Patient/Provider targeted interv	Young Adults, Healthcare Providers	VI: 75% increase in HPV vaccine doses in the 2018 vs. 2019 spring semester; a 77% increase in doses for 18–26 year-olds, across the two semesters	NR	F: Brief, relatively low cost intervention B: NR
**Grandahl et al., 2016 ** [48]	Sweden	Experiment	Single	X						Secondary Schools	Sample: Upper Secondary School Students—751 total, 394 intervention and 357 control Race: NR Gender: *Intervention:* Female 61.4%, Male 38.6%; *Control:* Female 41.6%, Male 58.4% Age: M = 16.1	Control: General information, including those on sexual health Intervention: 1 h face-to-face health interview with school nurse; leaflet. The intervention took about 30 min CG: Beh Interv Patient Decision Support	Adolescents	VC: intervention group increased vaccination status from 52.5% (before intervention) to 59% (after vaccination); no difference seen in the control group (60.9%) p-value = 0.02	Education: increased intention to use a condom with a new partner (1.751 higher points vs control group [p-value = 0.004]); intervention group perceived increased risk for HPV infection and HPV-related disease (1.675 points higher vs control group [p-value < 0.001]). increased condom use	F: School nurses had specialized and sensitive background/ training B: NR
**Gualano et al., 2019 ** [49]	Torino, Italy	Experiment	Single	X						University	Sample: 565 young adults Race: 93% born in Italy Gender: NR Age: M = 22.3	3 different kinds of informative material on HPV and vaccine: Journal article describing HPV infection, gynecologist video-interview, and institutional leaflet about HPV prevention CG: Information	Young Adults	NC	Knowledge: students showed increased knowledge (OR = 1.82, p-value = 0.02); students would strongly recommend HPV vaccination (OR = 3.45, p-value < 0.001)	F: NR B: NR
**Henrikson et al., 2018 ** [50]	Northwest, USA	Experiment	Single	X						GHC Primary care clinics	Sample: 1805 children (plus subset of 50 parents) Race: White 1049, African American 104, Hispanic 29, Asian 265, Native American 29, and Unknown 148 Gender: *Intervention Group*: Female 48.9%, Male 51.1%; *Control Group:* Female 46.8%, Male 53.3% Age: 10–12	An outreach letter and brochure recommending HPV vaccination followed by automated HPV vaccine reminders CG: Beh Interv Providers	Children	VI: rates of VI within 120 days of randomization was higher in the intervention group (23.6% and 18.8%, p-value-0.04) VC: vaccine completion during the study period was higher in the intervention group vs control group (10.3% vs 6.8%, p-value = 0.04)	NR	F: 74% parents had already decided about the vaccine B: Parents recall about receipt of the letter/IVR call, improving timing and # of reminders, use different mode of reminders
**Hofstetter et al., 2017 ** [51]	New York, USA	Experiment	Single	X						NR	Sample: parents and adolescents aged 11–17 years with Chronic Medical Conditions; 295 adolescents and parent, 71 providers Race: Latino 239 (81.9%), Non-Latino Black 38 (13%), Non-Latino White 5 (1.7%), and Other/multiracial 10 (3.4%) Gender: Female 45.8%, Male 54.2% Age: 11–17	Two types of text messages: Plain text message reminder Text message with educational content CG: Beh Interv Patient Decision Support	Children	VI: No difference between control and intervention group at 4 weeks, 12 weeks, or 24 weeks (p-values = 0.13, 009, 0.20)	Education: more adolescents received any needed vaccine for the reminder arm by 4 weeks (31.9% vs 22.7%), but not by 12 or 24 weeks’ Fewer adolescents in the plain vs educational reminder arm that had a missed vaccination opportunity by 4 weeks (10.9% vs 41.3%), but not by 12 or 24 weeks	F: NR B: NR
**Joseph NP et al., 2016 [**52**]**	Urban Area, USA	Experiment	Multi		X	X				Large urban hospital	Sample: 200 (100 mother/daughter dyads-50 per study arm) Race: 100% Haitian/African American (50% Haitian American, 50% African American) Gender: 100% Female Age: Mothers: M = 41; Daughters: 9–17	The BNI was administered to mothers by a trained intervention provider (10–20 min); components: mothers sharing own experience with the impact of HPV; assessment of advantages and disadvantages of vaccination to help resolve ambivalence while increasing self-efficacy about vaccine decisions CG: Beh Interv Patient Decision Support	Adolescents, Parents	VI: No significant difference between intervention and control group (56% vs 51%, *p* = 0.47) VC: No significant difference between intervention and control groups (21% vs 16% for second dose, *p* = 0.29 and 10% and 7% for third dose, *p* = 0.4)	Knowledge: increased knowledge about HPV among mothers in intervention group (pre/post mean score of 5 to 10 out of possible 11) and significantly higher mean knowledge scores (10 vs 6)	F: NR B: NR
**Juraskova et al., 2011 ** [53]	Sydney, Australia	Experiment	Single	X						University of Sydney	Sample: 159 students who had not already received the HPV vaccine Race: NR Gender: 100% Female Age: 17–26, M = 19	Cervical Cancer (CC) condition: information of a vaccine that protects against cervical cancer; CC + GW (genital warts) group: information that the vaccine protects against cervical cancer and genital warts CG: Information	Young Adults	VI: of those surveyed at 2 months, 44% of the participants in the CC and GW group and 32% of participants in the CC group had received HPV vaccination at follow-up; no significant association between groups and follow-up vaccination behavior (37%, *p* = 0.56)	Acceptability: barriers (*p* = 0.029) and benefits (0.001) independently predicted HPV vaccination intention; susceptibility (*p* = 0.023) and benefits (0.033) independently predicted HPV	F: NR B: NR
**Juraskova et al., 2012 ** [54]	Sydney, Australia	Experiment	Single	X						University	Sample: 159 students who had not already received the HPV vaccine Race: NR Gender: 100% Female Age: 17–26, M = 19	CC condition: information about the HPV vaccine and cervical cancer; CC + GW condition: additional information about genital warts CG: Information	Young Adults	NC	Education: no effect of information framing on intention to receive the HPV vaccine or vaccine uptake behavior at 2-month follow-up	F: NR B: NR
**Kaul et al., 2019 ** [55]	Rio Grande Valley, TX, USA	Quasi-Experiment	Multi	X				X		School district	Sample: 2,307 male and female middle school students at 3 schools Texas schools (1 school: intervention, 2 schools: controls) Race: NR Gender: *Intervention:* Female 51.53%, Male 48.47%; *Comparison:* Female 46.77%, Male 53.23% Age: *Intervention:* 9.9—14.4 *Comparison:* 9.94—14.22	Free HPV vaccination events were held and at each event: 2 tables-one with educational materials and another for the vendor that was contracted by the project to administer on-site vaccinations at the school CG: Information	Adolescents	VI: post intervention, the intervention school had higher initiation rates vs comparison schools (53.67% vs 41.56%, p-value < .001) VC: post intervention, the intervention school had higher completion rates vs comparison schools (28.36% vs 20.53%, p-value < .001); intervention school were > 3.6 × likely to newly initiate/complete HPV vaccinations vs comparison schools	NR	F: NR B: NR
**Kepka et al., 2011 ** [56]	WA, USA	Experiment	Single	X						Local health fairs and community events	Sample: 88 Hispanic parents or guardians of daughters aged 9–17 Race: 100% Hispanic/ Latin American Gender: Female 88.64%, Male 11.36% Age: 22–62, M = 39.9 (SD = 8.8)	• Intervention: HPV vaccine radionovela included about 5 min of typical Spanish radio programming, the HPV radionovela which was also 5 min in length, and then another 3 min of typical Spanish radio programming • Control: typical Spanish radio programming CG: Beh Interv Patient Decision Support	Children	NC	Knowledge: intervention group scored significantly higher on six knowledge and belief items more likely to confirm than control group parents	F: NR B: NR
**Kester et al., 2014 **[57]	IN, USA	Experiment	Single	X						Black and minority health fair	Sample: 131 female and male young adults Race: Non-Hispanic Black 77%, Non-Hispanic White 11%, and Other (mostly multi-racial individuals) 12% Gender: Female 70%, Male 30% Age: 18–26, M = 21.85	A 5–10 min small group presentation in the areas of HPV infection, detection, treatment and prevention CG: Information	Young Adults	NC	Knowledge: intervention group had higher HPV knowledge scores (Mean: 9.1) vs. control group (Mean: 7.0, F: 22.53) Intention: among unvaccinated (*n* = 79), intervention group had higher HPV vaccination intent (86%) vs. control group (67%) (OR = 3.09)	F: NR B: NR
**Kim & Nan, 2016 ** [58]	Eastern, USA	Experiment	Single	X						Large university	Sample: 416 undergraduate students Race: White 57.2%, Asian 19.5%, Black 16.3%, Hispanic 6.3%, and Other 0.7% Gender: Female 33.7%, Male 66.3% Age: M = 20.05	A mock health message promoting HPV vaccination: Either present oriented or future oriented CG: Information	Young Adults	NC	Intention: those with high consideration of future consequences (CFC) reported stronger intentions when the vaccine was offered for free (*p* = .05) compared to the future-oriented messages	F: NR B: NR
**Kumar et al., 2019 ** [59]	San Diego County, CA, USA	Quasi-Experiment	Single							Pediatric practices	Sample: 96 providers at 6 sites Race: NR Gender: NR Age: NR	20-min training video targeting barriers to strong provider recommendation of the human papillomavirus (HPV) vaccine CG: Behav Interv Providers	Providers	NC	Education: significant improvements in multiple areas; areas were knowledge of HPV-related disease burden, changes in vaccine response with age, comfort with counseling vaccine-hesitant parents	F: NR B: NR
**Kwang et al., 2016 ** [60]	Malaysia	Quasi-Experiment	Single	X						Local university students	Sample: 580 pre-university Malay students Race: Malays 94.7%, Others 4.5%, Chinese 0.7%, and Indian 0.2% Gender: Female 58.4%, Male 41.6% Age: 18–25	Intervention: Information leaflet Control group: No information CG: Information	Young Adults	NC	Knowledge: number of students with poor knowledge reduced from 48.3% to 29.3%	F: NR B: NR
**Lee et al., 2018 ** [61]	Lowell, MA, USA	Experiment	Single					X		NR	Sample: 18 Khmer American mother and daughter dyads Race: 100% Khmer Race: NR Gender: 100% Female Age: 14–17	Mother- daughter dyads: a 26 min video entitled “Save My Daughter from Cervical Cancer” CG: Beh Interv Patient Decision Support	Adolescents, Parents	VI: no difference in VI between intervention and control groups	Knowledge: daughters in intervention group reported higher intention to receive HPV vaccination within one month vs control group (4 vs 1)	F: NR B: Communication between mothers and daughters and between researchers and participants, conflicted relationships between mothers and daughter
**Lefevere et al., 2016** [62]	Flanders, Belgium	Quasi-Experimental	Single	X						NACM member girls	Sample: 221 (intervention) and 243 (control) for the personal information campaign and 629 (intervention) and 5,322 (control) for the combined personal information and financial incentive campaign Race: NR Gender: 100% Female Age: 12–17	Vaccine Reimbursement Campaign: a letter; Leaflet with information on HPV, cervical cancer and the role of the HPV vaccine in the prevention of cervical cancer CG: Beh Interv Patient Reminder	Adolescents, Young Adults	VI: intervention vs control group for older girls (64.6% vs 42.8%) one year after the campaign; for younger girls, intervention vs control (78.4% vs 68.1%)	NR	F: NR B: NR
**Lennon, et al., 2019 ** [63]	Milwaukee, WI, USA	Quasi-Experiment	Multi		X			X		NR	Sample: 118 adolescent parent dyads Race: 100% Black/ African American Gender: *Adolescents*: Female 57%, Male 43; *Parents*: Female 92%, Male 8% Age: 13–17	Communication tools that were designed as part of CHIMC-TCI! dissemination plan included: 1) CHIMC-TCI! Parent Toolkit, 2) 4 module, interactive eLearning Café accessible on the website, 3) multimedia campaign, 4) Postcards were mailed to families to remind parents/caregivers of their child’s/adolescent’s immunization status CG: Beh Interv Patient Reminder	Adolescents	VC: increase from 30 (25%) at enrollment to 54 (46%) at study completion (*p* = 0.004)	Knowledge: those that completed the vaccine were more confident with safety of childhood immunizations (97%), vs those that did not complete the vaccine (79%)	F: exposure to multiple interventions B: NR
**Lin et al., 2019 **[64]	Colombia, Mexico and Panama	Experiment	Single	X						NR	Sample: 74 girls aged 4 to 6 years of age at the time of first vaccination Race: 100% American Hispanic/ Latino Gender: 100% Female Age: 4–6, M = 4.3	Intervention: received 2 doses of AS04-HPV-16/18 vaccine (Cervarix, GSK, Belgium) at months 0 and 6 CG: Environ Small Policy	Children	NC	Efficacy: over 36 months there were no withdrawals due to adverse events	F: NR B: NR
**Liu et al., 2019 ** [65]	Chengdu, Sichuan Province, China	Experiment	Single	X						NR	Sample: 1675 adolescents in mainland China Race: Han 1579 (94.3%), Others 49 (2.9%), and Unknown 47 (2.8%) Gender: Female 45.9%, Male 52.8%, and No Response 1.3% Age: 10–14	Intervention: PowerPoint- orientated health education CG: Information	Adolescents	NC	Acceptability: willing to be vaccinated before 55.2% to 88.4% after intervention compared to control group; intervention group was more aware of cervical cancer, HPV and the vaccines	F: NA B: Study provides information to policy makers on how important health education is
**Malo et al., 2016 ** [66]	USA	Non- Experiment	Multi	X		X				National surveys	Sample: Parents, *n* = 1504. Primary care physicians, *n* = 776 Race: Parents only: White Non-Hispanic 1,058 (70%), Black Non-Hispanic 135 (9%), Other Non-Hispanic 99 (7%), and Hispanic 212 (14%) Gender: *Parents:* Female 56%, Male 44%; *Parent's children:* Female 49%, Male 51%; *Physicians:* Female 32%, Male 68% Age: NR	Parents were randomly assigned to panels (Panel A, Panel B, Panel C), each of which included two brief messages and three longer messages in a random order CG: Patient/Provider Targeted Interv	Parents, Healthcare Providers	NC	Education: parents unlikely to vaccinate were in favor of messages with information about HPV infection being common, cancers caused by HPV, and HPV vaccine effectiveness; endorsement of all 15 messages was higher among parents whose children had received HPV vaccine (all *p* < .05); about 39% of physician favored the brief messages	F: NR B: NR
**Malo et al., 2018 ** [67]	NC, USA	Experiment	Multi			X	X			Clinics	Sample: 83 vaccine-prescribing and 59 non-prescribing clinicians Race: NR Gender: *Announcement arm:* Female 72%, Male 28%; *Conversation arm:* Female 66%, Male 34% Age: NR	Intervention: four 1-h trainings to vaccine-prescribing clinicians and other staff using a standardized script and PowerPoint slide set CG: Beh Interv Providers	Healthcare Providers	NC	Education: amount of time providers reported needing to discuss HPV vaccination with parents decreased for both trainings from pre-training to 1-month follow-up (mean = 3.8 vs. 3.2 min, *p* = .01, d = .28)	F: NR B: Recommendation discussions still take more time compared to discussing other types of vaccinations (i.e.meningitisand Tdap)
**Mantzari, Vogt, & Marteau, 2015 ** [68]	Birmingham, England, UK	Experiment	Single	X						NR	Sample: 500 girls registered with general practitioners Race: NR Gender: 100% Female Age: 16–18	Financial incentive of $65 for receiving the full 3 shot HPV vaccination series CG: Beh Interv Patient Decision Support	Young Adults	VI: increased initial uptake of vaccination program by about 10% in both first time invitees (OR = 1.63) and previous non-attenders (OR = 0.611) VC: combination of financial incentives and text messages increased completion of vaccination program by about 10% in both first-time invitees (OR = 2.152) and previous non-attenders (OR = 4.283)	Acceptability: effect of the intervention uptake of first and third vaccinations was not impacted by social deprivation in either first time invitees (first vaccination: OR = 0.985, third vaccination: OR = 1.002), or previous non-attenders (first vaccination: OR = 0.998, third vaccination: OR = 1.007)	F: NR B: technology barriers for reminders
**Marchand-Ciriello** **, ** **Foustoukos, & Fantasia, 2020 **[69]	Northeastern MA, USA	Quasi-Experiment	Multi			X	X			Pediatric practices	Sample: 13 pediatric providers and 520 males who had not received the initial HPV vaccine Race: NR Gender: *Patients:* 100% Male; *Pediatricians:* Female 4 (31%), Male 4, (31%); *Nurse practitioners:* females 6 (38%) Age: *Patients:* 11–21; *Providers:* 31–73	An electronic medical record prompt, educational presentation, monthly e-mail updates to providers CG: Beh Interv Providers	Healthcare Providers	VI: rates increased by 6.5%; VI rates were higher among adolescent males with publicly funded health insurance (49.6% vs 39.7%)	Education: EMR prompt was the most effective tool reported by providers (9 out of 12)	F: NR B: NR
**McGlone et al., 2017 ** [70]	Assume, and Houston, TX, USA	Experiment	Single	X						NR	Sample: 167 Spanish-speaking Hispanic/Latina mothers Race: 100% Hispanic Gender: 100% Female Age: NR	Participants received a Spanish text message on their phones described by the survey administrator as a reminder the clinic might send to arrange a vaccination appointment CG: Beh Interv Patient Reminder	Parents	NC	Acceptability: reminder messages that framed virus transmission as an action made mothers perceive the threat as more severe (F (1,163) = 13.66, *p* < .001, d = .41)	F: NR B: NR
**McLean et al., 2017 ** [71]	Central, Northern and Western WI, USA	Experiment	Multi			X	X			Pediatric/ family practice	Sample: 9 clinics (6 pediatric and 3 family practice/other) that see patients aged 11–17 Race: NA Gender: *Intervention:* Female 49%, Male 51%; *Control:* Female 49%, Male 51% Age: NA	Provider and staff education; quarterly feedback to providers; reminder and recall notices CG: Beh Interv Providers	Healthcare Providers	VC: significant increase (32.0% before to 52.7% after)	Education: HPV vaccine coverage in the intervention increased from 41 to 59%, significantly greater than in the control (32% to 45%, *p* = .0002). The increase occurred after completion of provider and staff education and a patient reminder/recall system (*p* = .004)	F: NR B: NR
**McRee et al., 2018 ** [72]	USA	Experiment	Single	X						National sample	Sample: 150 youth, Gay and Bisexual Men (YGBM) Race: Non-Hispanic White 58%, Non-Hispanic Black 13.5%, Hispanic, 22% Gender: 100% Male Age: 18–25	A web-based intervention, Outsmart HPV, to promote HPV vaccination among YGBM Control: standard HPV vaccination information (control) Intervention: population-targeted, individually-tailored content CG: Information	Young Adults	NC	Education: intervention group had a stronger perception that men who have sex with men are at higher risk for anal cancer relative to other men (b = 0.34); greater HPV vaccination self-efficacy (b = 0.15); and fewer perceived harms of HPV vaccine (b = -.34) on posttest surveys (all *p* < .05)	F: NR B: NR
**Meyer et al., 2018 ** [73]	Rochester, MN, USA	Quasi-Experiment	Multi			X	X			Retail Clinics	Sample: 3,234 eligible patients to receive HPV vaccine Race: NH White 2,66 (85.5%), NH Asian 87 (2.7%), NH Black 80 (2.5%), Hispanic 117 (3.6%), Other/unknown 184 (57%) Gender: Female 1,732 (53.6%), Male 1,502 (46.4%) Age: 9–26 M = 14.11	A 2- hour lecture for providers was carried out and an electronic point- of care prompt was introduced CG: Beh Interv Providers	Health Systems/ Organizations	VI: 2% of pre-prompt time period patients received a dose of vaccine; 12% of post-prompt patients received a dose of vaccine. The point-of-care prompt increased the median weekly HPV vaccination rate by 8.6 per 100 patient visits (*p* < 0.001)	Education: of patients reporting prompting, 97.5% stated it was convenient having HPV vaccine available, 91.6% stated it was helpful to be reminded during the visit, 94.6%	F: NR B: Clinicians that were hesitant about the HPV vaccine may have been less compliant to complete the point-of-care prompt
**Mohanty et al.**, **2018 ** [74]	Philadelphia, PA, USA	Non- Experiment	Single	X						Facebook	Sample: 152 adolescents that received HPV vaccine through 3forME Race: Black/African American 68 (45%), Hispanic 38 (25%), Asian 27 (18%), White 5 (3%), and Other 14 (9%) Gender: Female 53 (35%), Male 99 (65%) Age: 13–18	A Facebook campaign with six specific messages about HPV immunization: • Ran for two-week intervals • Reminder-recall letters were also sent to adolescents CG: Beh Interv Patient Reminder	Adolescents	VI: 73 (48%) adolescents received their first dose, 40 (26%) adolescents received their second dose VC: 39 adolescents received their third dose; 63 (41%)	Acceptability: few signed up for vaccine appointments through the Facebook page, only 2 signed up through the 3forMe website and did not receive reminder-recall letters	F: NR B: Participants comfort in receiving vaccines or services outside of their primary care practice
**Molokwu et al.**, **2019 ** [75]	El Paso, TX, USA	Non- Experiment	Multi	X				X		Community sites in El Paso County	Sample: 1,796 total (937 adults and 859 children) adolescents or parents of adolescents who had not completed the HPV vaccine series Race: Hispanic 1512 (97.4%), NH 40 (2.6%) Gender: Female 1148 (63.99%), Male 645 (36.01%) Age: 18–26 adolescents or parents of adolescents aged 9–17	Outreach education, navigation, and provision of vaccine CG: Patient/Provider Targeted Interv	Young Adults, Parents	VI: VI rate was 67.1%; significantly higher among adults (77.4%) vs children (55.8%); 90% of participants received at least one dose, and 55.5% received at least 2 doses VC: 39.8% and was low among adults (31.6%) vs children (48.7%)	Awareness: among adult participants, HPV awareness improved significantly from 62.7% to 87.6%	F: NR B: NR
**Morales-Campos, & Parra-Medina, 2016 **[76]	Cameron County, TX; Hidalgo County, TX, USA	Quasi-Experiment	Single	X						NR	Sample: 317 mothers of unvaccinated daughters aged 11–17, Race: 100% Hispanic Gender: Female 100% Age: M = 38	Community health workers and undergraduate peer educators were utilized to deliver education and navigation to mothers: • Education included a 1-h education session for each the mother and daughter • Mothers who attended education sessions received a community resource sheet listing clinics offering free or low-cost HPV vaccinations CG: Beh Interv Patient Decision Support	Parents	VI: No significant association between VI and HPV knowledge (OR = 0.91), HPV vaccine knowledge (OR = 0.94), and HPV vaccine self-efficacy (OR = 1.03). Insured mothers were 79% less likely to report their daughter initiated vaccine vs uninsured mothers (AOR = 0.29). Mothers that received the EMPH program were less than 2 × as likely to initiate HPV vaccine vs brochure-only group (AOR = 1.81) VC: no significant association between VC and HPV knowledge (OR = 0.96), HPV vaccine knowledge (OR = 0.98), and HPV vaccine self-efficacy (OR = 0.98)	Knowledge: no association between daughters’ vaccine completion and mothers’ HPV vaccine self-efficacy (AOR = 0.98), HPV knowledge (AOR = 0.95), and HPV vaccine knowledge (AOR = 1.05)	F: NR B: NR
**Nissen et al., 2019 ** [77]	SD, USA	Non- Experiment	Multi		X	X	X			Clinics	Sample: 39 clinics (7 family medicine clinics in year one, and an additional 32 primary care clinics in year 2) Race: NA Gender: Both were included but totals and percentages of genders were NR Age: Patients: 11–26	The multi-level intervention included: clients reminders (automated phone and mail), recall system vaccine education for providers and staff, and provider assessment and feedback re-education on standing orders CG: Patient/Provider Targeted Interv	Health Systems/ Organizations	VI: in year one, vaccine administration nearly doubled from 1,554 doses to 2,986 doses. In year 2, dose administration increased by 48.7% overall. Among clinics participating in both project years, rates of zero-dose vaccination dropped from 64.2% to 42%. Across all 39 sites in year 2 saw zero-dose vacicnations drop from 54% to 44.8% VC:13% increase in VC from project beginning to close	Education: across 7 sites in year one, 41,576 reminders were distributed (mail and phone reminders); in year 2 across 39 sites, 62,995 reminders were sent out	F: NR B: Change in vaccine dosage during the project from 3 doses to 2 for most adolescents
**Nwanodi, Salisbury, & Bay, 2017 ** [78]	USA	Experiment	Single	X						Online	Sample: 1109 (Young adults and parents of children) Race: NH White 727 (65.6%), Hispanic White 122 (11%), NH Black 108 (9.7%), Non-Hispanic Asian 76 (6.9%), NH mixed 25 (2.3%), NH Other 13 (1.2%), Hispanic Other 4 (0.4%) Gender: Female 633 (57.1%), Male 476 (42.9%) Age: 19–26; 27 +	Four components of the intervention: • 14-sentence information brief • counseling intervention: 14 sentence brief + 4.34-min audiovisual • counseling intervention: 14-sentence info brief + public health education handout (PHEH) • counseling intervention.: 14-sentence info brief + audiovisual + PHEH CG: Information	Young Adults, parents	NC	Knowledge: intervention raised knowledge of HPV vaccination purpose (*p* = 0.02) Acceptance: vaccination acceptance for seven items (*p* < 0.001 to *p* = 0.023)	F: NR B: NR
**Obulaney, Gilliland, & Cassells, 2016 ** [79]	Southeastern, TX, USA	Quasi-Experiment	Single	X						Faith- based clinics	Sample: 41 mothers at faith-based clinic setting Race: Non-Hispanic White 25.6%, Hispanic 67.4%, and Black 7% Gender: 100% Female Age: 28–56	A language- appropriate education session was offered: • Brochure • 11- minute video • Q&A session CG: Beh Interv Participant	Parents	VI: During 3 months prior to intervention, 3 vaccines given to 56 girls. During 3 months of the initiative, 22 vaccines given to 120 girls. Overall vaccine rate increased from 5.4% to 18%	Education: improvement in knowledge about risk and transmission of HPV from pretest (mean = 79.51%) to posttest (mean = 90.73%). Improvement in mothers’ intent to have daughters vaccinated (56% pretest vs 81% posttest)	F: NR B: lack of consistently available translators and participant literacy level
**Padmanabha et al., 2019 ** [80]	Mangalore, India	Non- Experiment	Single	X						Mangalore Medical School	Sample: 263 medical students Race: 100% Asian/ Pacific Islander Gender: 100% Female Age: 18–25	information session lasting for five minutes CG: Information	Young Adults	NC	Intention: 59% of previously unvaccinated participants stated they would definitely get vaccinated. 34% were unsure, and 7% were unwilling	F: NR B: NR
**Parra-Medina et al., 2015 ** [81]	Cameron County, Hidalgo County, TX, USA	Experiment	Single	X						NR	Sample: 372 mothers of daughters aged 11–17 who had not received HPV vaccine Race: 100% Hispanic/ Latin American Gender: 100% Female Age: M = 38.4	This was a culturally relevant cervical cancer prevention program (Entre Madre e Hija (EMH)) health education model: • separate groups for mothers (promotora-delivered-community health workers) & daughters (student peer educators) • Referral and navigation support from a *promotora*-community health worker • Those that declined participation in EMH received the brochure only CG: Beh Interv Patient Decision Support	Parents, Adolescents	VI: 84% initiatied vaccine. No differences between EMH program and brochure-only parents VC: EMH more likely to complete the series vs brochure-only (AOR = 2.24); those whot were employed (AOR = 0.45) and insured (AOR = 0.36) were less likely to complete vaccine series	NR	F: NR B: NR
**Paskett et al., 2016 ** [82]	Appalachia, OH, USA	Experiment	Multi	X		X	X			Participating counties or clinics	Sample: 337 parents of a daughter aged 9–17 that had not received HPV vaccine and 119 providers from 24 clinics Race: Providers: White 95%, Other 5%, Hispanic 0.8%, NH 99.2% Parents: NH White 98.5%, and Other 1.5% Gender: Providers: Female 92.4%, Male 6.7%; Parents: Female 92.3%, Male 7.3% Age: Providers: M = 49.2; Parents: M = 43.5	Parent-level Intervention: • mailed a packet with an educational brochure, DVD video about HPV and HPV vaccination, a magnet reminder to get the 2nd and 3rd HPV vaccine shot Provider-level Intervention: • 1-h PowerPoint presentation and handouts on the HPV vaccine, focusing on current evidence-based HPV vaccine information and strategies designed to assist physicians in discussing HPV vaccination with parents Clinic-level Intervention: • information about HPV vaccination was visible and readily available CG: Patient/Provider Targeted Interv	Healthcare Providers, Parents, Health Systems/Organizations	VI: 7.7% of daughters in intervention group received first shot of vaccine within 3 months vs 3.2% of daughters in comparison group; By 6 months, 13.1% of daughters in intervention group received first vaccine vs 6.5% in comparison group	Knowledge: provider knowledge about HPV increased from baseline (4.4 correct answers) to post education (4.9 correct, *p* < 0.001) Behavior: Provider ability to talk to parents and patients about HPV vaccine ( intervention) was similar at baseline (89% for parents/patients; 12 months (83% patients and 92% patients)	F: NR B: NR
**Patel et al., 2012 ** [83]	MI, USA	Experiment	Single	X						University gynecology clinic	Sample: 256 females attending a university health service gynecology clinic Race: White 172 (67.2%), Asian 35 (13.7%), African American 29 (9%), Other 13 (5.1%), Mixed 13 (5.1%), and Hispanic 12 (4.7%) Gender: 100% Female Age: 18–26	Intervention group: • The study coordinator discussed in detail a “HPV and Vaccination” fact sheet. It contained bulleted information on HPV and its link to cervical cancer, ways to reduce risk of HPV infection, quadrivalent vaccine administration, cost, and insurance coverage, who should get the vaccine, and contraindications to the vaccine • Approximately two weeks after their clinic visit, they were mailed a packet containing a reminder letter describing the HPV vaccine and how to schedule a vaccine appointment along with another copy of the “HPV and Vaccination” fact sheet CG: Beh Interv Patient Reminder	Young Adults	VI: 5.5% participants received at least one HPV vaccine dose within six months of study enrollment. The education-based intervention was not significantly associated with HPV vaccine uptake (RR = 0.84)	Acceptability: 41% of participants indicated desire to undergo vaccination, 31.3% did not intend to get it, and 26.2% were unsure	F: NR B: NR
**Porter et al.**, **2018 ** [84]	USA	Experiment	Single	X						NR	Sample: 762 parents of girls aged 9–17, Race: White 74.7%, African American 6.4%, Asian 5.5%, Hispanic 4.3%, American Indian/Alaska Native Hawaiian/Pacific Islander 5.7%, and Other/Multi-race 3.4% Gender: Female 70.5%, Male 29.5% Age: CDC message: 39.5 Cervical Cancer Message: 39.2 Control message: 40.2	Comparing three messages: • A CDC HPV message • The cancer-salient message designed by the research team-framing it as protection against cervical cancer • A non-vaccine control message (about bird feeding) CG: Information	Parents	NC	Intention: Odds of reporting intent to vaccinate among cervical cancer message arm were 1.13 × the odds among control arm Intent to vaccinate was not statistically significant different between CDC message and control arm (OR = 1.25)	F: NR B: NR
**Poscia et al., 2019 ** [85]	Lazio, Basilicata, and Sicily, Italy	Experiment	Single	X						Secondary Schools	Sample: 755 Italian secondary school students at 2 schools Race: NR Gender: Female 48.1%, Male 51.9% Age: 11.3–13.3	Each class received a 90 min health promotion intervention, which includes: • A theoretical introduction and a second part more interactive using role-play • Students’ parents received informed consent paperwork and an invitation to a meeting with the project team • Students and parents then received at least one day in a clinic to carry out the recommended vaccinations for adolescents CG: Patient/Provider Targeted Interv	Adolescents	VI: After 8 months, more students received the HPV vaccine in the intervention school vs control school (30.5% vs 13.8% of females)	Other vaccines: The intervention school had higher vaccine rates for Meningococcal B, but lower ones for the 4^th^ dose of dTap. After 8 months, there was higher vaccine rates for Meningococcal C (6% vs 2%) and Meningococcal B (14.7% vs 0.3%) in the intervention vs control schools	F: NR B: NR
**Pot et al., 2017 ** [86]	Netherlands	Experiment	Single		X					Dutch vaccination register (Praeventis) and three Web-based panels	Sample: 8,062 Dutch mothers of daughters born in 2002 Race: NR Gender: 100% Female Age: 43.64 (SD = 4.25)	A computer-tailored intervention with virtual assistants providing mothers of girls to be invited with tailored feedback on their decision making about the HPV vaccination; website contained components that reviewed HPV information and risk of contracting HPV infection CG: Information	Parents	VI: There was no effect on uptake of the vaccine (*p* = 0.6)	Intentions: significant positive effect on informed decision making, decisional conflict, and nearly all determinants of HPV vaccine uptake (*p* < 0.001)	F: NR B: NR
**Reno et al., 2018 ** [87]	Central CO, USA	Quasi-Experiment	Single		X					Pediatric and family clinics	Sample: 8 clinics with providers who see adolescents & may interact with vaccine-hesitant parents Race: NR Gender: NR Age: NR	Healthcare providers and staff received communication training that included Motivational Interviewing (MI) techniques in the form of three parts: • A 40-min background video completed on own time • A 1-h in-person training session focused on describing and demonstrating techniques to use with vaccine hesitant parents • A 1-h in-person training with role playing of MI techniques CG: Beh Interv Providers	Healthcare Providers	NC	Education: Majority of providers believed MI was most effective when trying to educate and lead vaccine-hesitant parents when comparing to other intervention tools	F: NR B: time at patient visit to start a talk using MI, not being able to fit MI into their current workflow, prioritizing other health issues
**Rhodes et al.**, **2017 ** [88]	Missouri, USA	Non- Experiment	Single	X						Schools	Sample: 440 lead school nurses Race: NR Gender: NR Age: NR	An online, interactive module on HPV, HPV as cancer prevention and CDC recommendations for providers and prevention recommendations CG: Information	Healthcare Providers	NC	Knowledge: sign differences in mean scores between the pre-test (M = 6.28) and the posttest (M = 9.15, t (334) = –16.337; p, .05)	F: NR B: NR
**Richman et al., 2016 ** [89]	NC, USA	Experiment	Single	X						College	Sample: 264 male and female US college students 18–26 years old who were receiving HPV vaccine dose 1 Race: White 140 (54%), Black 69 (26%), and Other 53 (20%) Gender: Female 62%, Male 38% Age: 18–26	7 electronic messages, once per month across 7 months (4 health education messages about HPV and the HPV vaccine, 2 appointment reminder messages, and 1 message asking participants to take the follow-up survey); standard-of-care at the student health center (paper card with next appointment date); participant incentives CG: Beh Interv Patient Reminder	Young Adults	VC: HPV vaccine completion across groups were not significantly different for HPV dose 2 (53% vs 52%) and dose 3 (34% vs 32%); biggest predictor of HPV vaccine completion was female gender	Knowledge: mean knowledge score at follow-up for intervention group was significantly higher (mean score = 93%) than at baseline (mean score = 87%, *p* = 0.01); no significant differences in knowledge were found for the control group	F: NR B: changed to offer the vaccine at no cost to study participants; unreachable participants or didn't check their school email
**Richman et al., 2019 ** [90]	Pitt and Greene counties, NC, USA	Experiment	Single		X					NR	Sample: 257 parent–child dyads were included with 129 dyads randomized to the intervention group and 128 randomized to the control group Race: Black 60%, Hispanic 28%, and White 9% Gender: *Parents:* Female 88%, Male 12%; *Children*: Female 46%, Male 54% Age: *Parents:* 19–69; *Children:* 9–17	Parents-child dyads received seven electronic messages (in English/ Spanish based on preference) once per month across seven months Parents-child dyads in the control group: received standard-of-care at the clinics CG: Beh Interv Patient Reminder	Parents, Adolescents	VC: Rates of completion for dose 2 and 3 were similar for both intervention and control groups (65% for both dose 2, and 35% vs 30% for dose 3, respectively)	Knowledge: mean knowledge change between baseline and follow-up was higher in the intervention group (0.36) vs control (0.21), not statistically significant Acceptability: those recommended to receive the vaccine were 1.8 times more likely to complete the series	F: NR B: NR
**Rickert et al., 2015 ** [91]	Galveston County, TX, USA	Experiment	Single		X					Teen Health Center	Sample: 445 parents of male and female adolescents (ages 11 to 15 y who had not previously received the HPV vaccine) Race: White 30.6%, African American 27.9%, Hispanic 39.8%, Other 1.8% Gender: *Parents:* Female 87.4%, Male 12.6%; *Children:* Female 33.9%, Male 66.1% Age: *Parents:* M = 41.8 *Teens:* M = 13.5	Parent Health message intervention: • Initiated with the use of rhetorical questions (RQ) and then with the one- or two-sided message and was not blinded to the research assistant • Participants randomized into one of 4 conditions: 1) rhetorical questions (RQ) plus one sided message 2) RQ plus two-sided message 3) no RQ plus one-sided message, 4) no RQ plus two-sided message CG: Beh Interv Patient Decision Support	Parents, adolescents	VI: 34% of adolescents received their first dose VC: 67% completed the series	Intentions: RQ component of the intervention increased intention to vaccinate (RR = 1.45), but did not affect vaccine initiation or completion	F: NR B: NR
**Rockliffe et al.**, **2018 ** [92]	London, England	Experiment	Single				X			Secondary schools with year 8 students	Sample: 181 female adolescents in secondary schools in Year 8, and 61 parents, and 6 school staff members Race: Parents: Non-White British 49%; Adolescents: NR Gender: *Adolescents:* 100% Female; *Parents:* Female 64%, Male 36% Age: NR	Schools in the incentive intervention arm: • Provided HPV vaccination consent forms to year 8 girls to bring home and get signed by parents • Those who returned the waiver, regardless of ‘yes’ or ‘no’ to vaccination, were entered into a drawing for one of several £50 gift cards CG: Beh Interv Patient Decision Support	Parents, Adolescents	NC	Acceptability: there was a mix of positive, negative, and ambivalent responses about the use of the incentive to encourage HPV vaccination consent forms returned, both by the adolescents and their parents	F: NR B: NR
**Roussos-Rosset al.**, **2017 ** [93]	North Central FL, USA	Quasi-Experiment	Single	X						Women’s advantage meeting at University of Florida, Restoring Joy Church	Sample: 100 participants from 2 community outreach educational seminars at 2 venues Race: White 38%, Black 25%, Hispanic 2%, Other 12%, and Not stated 23% Gender: Female 64%, Male 13%, and Not Stated 23% Age: 18–65	a 30-min community outreach educational seminar CG: Information	Parents,, Adolescents, Young Adults	NC	Knowledge: significant difference in pre-and post-test knowledge Willingness: participants were more willing to receive vaccine or allow child to get it at posttest (66.2% vs 49.5%, pre vs posttest)	F: NR B: NR
**Sadoh et al., 2018 ** [94]	Benin City, Nigeria	Non- Experiment	Single	X						Secondary Schools	Sample: 1337 female students from four secondary schools in Benin City, Nigeria Race: NR Gender: 100% Female Age: 9–17	Lecture emphasized key information on cervical cancer while each student was given a flier containing the key information. Within two weeks, each student delivered a mini lecture on the subject to her classmates using the flier as a guide and to emphasize key points CG: Information	Adolescents	NC	Awareness: significant difference in awareness of cervical cancer pre-training (14.8%) vs post-training (97.8%, *p* < 0.0001) Knowledge: mean score was highly significant, 60.39 ± 9.75 vs the pre-training mean score (*p* < 0.0001)	F: NR B: NR
**Schnaith et al., 2018 ** [95]	Twin Cities and Duluth, MN, USA	Quasi-Experiment	Single	X						Medical School	Sample: 132 medical school students at University of Minnesota 101 completed pre and post intervention surveys Race: NR Gender: Female 66%, Male 33%, and did not specify 1% Age: NR	Participated in an HPV vaccination curriculum consisting of: • a lecture • video • role-play simulation CG: Beh Interv Providers	Healthcare Providers	NC	Education: student awareness of HPV vaccine benefits increased by an average of 0.82 points Behavior: student comfort talking to vaccine hesitant parents increased by 1.37 points	F: NR B: NR
**Shah et al., 2019 ** [96]	USA	Experiment	Single	X						Address- based sampling	Sample: 1196 parents of children aged 9–17 who had not initiated HPV vaccine series or only received first dose Race: Non-Hispanic White 70%, Non-Hispanic black 9%, Non-Hispanic multiracial or other 7%, Hispanic 14% Gender: Female 54%, Male 46% Age: M = 42.7 (SD = 8.1)	There were 2 different video-messaging experiments: • first: parents were randomly assigned to conditions by employing different vaccine recommendation strategies • second, parents were randomly assigned to messages where questions/concerns about HPV vaccine topics were answered CG: Information	Parents	NC	Vaccine efficacy: confidence in HPV vaccine increased following messages about HPV vaccine (b = 0.13), messages on cancer prevention (b = 0.11); less confident following messages that expressed urgency (b = -0.06)	F: NR B: NR
**Staples, Wong, & Rimmel, 2018 ** [97]	HBCUs in the Southeast US, Hampton University, Marshall University, West Virginia State University, NC Central University, USA	Quasi-Experiment	Single	X						Colleges	Sample: 57 female HBCU students Race: 100% Black/ African American Gender: 100% Female Age: reported as under 17, 18–20, and > 21	1-h lecture which included: • Power Point presentation • female body diagrams • topic-specific medical instruments including plastic speculums and Pap brushes CG: Beh Interv Patient Decision Support	Young Adults	VI: 29 participants (53%) reported at least starting the series, the remainder were either unsure of their vaccination status (*n* = 8, 15%) or had never been vaccinated (*n* = 18, 33%). VC: A total of 24 participants (42%) reported completing all 3 doses, which yields a series completion rate of about 83%	Education: significant increase (74% vs 91%) for the intervention; following intervention, 94% confirmed they will get regular pap smears, and 87% planned to get HPV vaccine	F: NR B: NR
**Staras et al.**, **2015 **[98]	Gainesville, FL, USA	Quasi-Experiment	Multi	X			X			Florida Medicaid or Children’s Health Insurance Program	Sample: 2773 girls and 3350 boys without prior HPV vaccine claims in Florida Medicaid or Children’s Health Insurance Program Race: Non-Hispanic White 47%, Non-Hispanic Black 26%, Hispanic 14%, and Other 13% Gender: Female 2773 (45%), Male 3350 (55%) Age: 11–17	Adolescents were assigned to one of four study arms: 1) postcard campaign, (2) in-clinic HIT system, (3) postcard campaign and in-clinic HIT system, and (4) usual care CG: Patient/Provider Targeted Interv	Adolescents	VI: 5% of adolescents initiated the series; odds of VI increased with the postcard campaign (60% among girls and not significantly among boys), with the HIT system (50% among girls and 40% among boys), and with the combined postcard campaign and HIT system (140% among girls and 60% among boys)	Information seeking: majority of parents (91% of boys and 80% of girls) sought additional information about the vaccine after receiving the postcard	F: NR B: difficulties with incorporating the HIT system into the clinic workflow
**Stern et al., 2013 **[99]	9 Planned Parenthood health centers located in NC, UT, AZ, WA, CO, CA; and one hospital family planning clinic located in IL, USA	Experiment	Single	X						Family planning facilities	Sample: 365 women from 10 reproductive health centers Race: White 57%, African American 11.5%, Latin 21.6%, Asian 4.7%, and Other 5.2% Gender: 100% Female Age: 19–26	An automated system to remind participants when their next HPV vaccine dose was due. Participants could receive their reminders via text, email, phone, private Facebook message, or standard mail CG: Beh Interv Patient Reminder	Young Adults	VI: not significant difference on returning for second dose (40.6% intervention group vs 40% control group, *p* = 0.915) VC: reminder system messages did not increase VC (17.2% for intervention vs 18.9% in control, *p* = 0.881)	NR	F: NR B: Participants not aware that they were eligible for financial assistance
**Underwood et al., 2015 ** [100]	Eastern GA, USA	Experiment	Multi	X	X					Middle/ High Schools	Sample: 686 parents from 11 schools Race: Adolescents: White 16.2%, African-American 74.9%, and Other 8.9% Gender: Adolescents: Female 53.1%, Male 46.9% Age: M = 14.2	Each school was randomly assigned to one of 3 study arms: • Arm 1) no intervention (control) • Arm 2) an educational brochure about adolescent vaccines mailed home for parents (parent-only) • Arm 3) curriculum implemented by science teachers in classrooms of adolescents, plus educational brochures used in arm 2 (parent and adolescent) CG: Beh Interv Patient Decision Support	Parents, Adolescents	VI: A report of VI among parents increased during the final follow-up (aOR = 1.76); parents with more positive attitudes about HPV vaccine were more likely to begin VI vs parents with lower HPV attitudinal scores (aOR = 2.08). Female adolescents had 3 × odds of beginning VI (aOR = 3.0) VC: Females had greater odds of VC vs males (aOR = 2.1). Parents with higher HPV attitude and belief scores had higher odds of child completing series vs parents with lower scores (aOR = 1.2)	Attitudes: parents whose child received one HPV dose had higher HPV attitude scores compared to those without a HPV dose (mean = 4.5 vs mean = 3.2)	F: NR B: NR

Note. *NC* Not collected, *NR* Not Report. Levels: *Ind* Individual, *Inter* Interpersonal, *Prov* Provider, *Org* Organization/Clinic, *Comm* Community, *Pol* Policy. Race: *NH* non-Hispanic. Community Guide interventions: *Beh Interv Patient Decision Support* Behavioral intervention patient decision support, *Beh Interv Patient Reminder* Behavioral intervention patient reminder, *Beh Interv Providers* Behavioral intervention provider-targeted, *Patient/Provider Targeted Interv* Patient and provider targeted intervention, *Environ small policy* Environmental intervention small policy, *VI* Vaccine initiation, *VC* Vaccine completion, *QI* quality improvement

### Study setting and design

Of the 79 intervention articles, 57 (72.2%) were conducted in the U.S. Other studies were conducted in Europe (*n* = 10, 12.7%), Africa (*n* = 4, 5.1%), Asia (3, 3.80%), Australia (3, 3.80%), Central/South America (1, 1.27%), and Canada (1, 1.27%). Forty-five studies (57.0%) employed an experimental design, 18 (22.8%) used a quasi-experimental design, and 16 (20.3%) employed a non-experimental design.

#### Setting and population focus

Intervention settings included clinics (32, 40.5%), schools (26, 32.9%), communities (10, 12.7%), an organization (1, 1.3%), a health insurance system, and online (10, 11.4%). Study samples ranged from 36 to 8,062.

Of the 79 studies, most interventions targeted adolescents only (39 studies, 49%) [22, 25, 27, 29, 31, 32, 34–36, 40, 43, 44, 46, 48, 50–55, 60–65, 68, 69, 72–75, 85, 90–92, 94, 98, 100], of which 15 (38%) included girls only, 17 (44%) included both boys and girls, 3 (8%) included boys only, and 4 (10%) did not report. Other interventions focused on young adults ages 18–34 years (20 studies, 25%) [22–26, 28, 34, 38, 47, 49, 57, 58, 69, 73, 78, 83, 89, 93, 97, 99], parents (27 studies, 34%) [25, 33, 41, 43, 45, 50–52, 56, 61, 63, 66, 70, 75, 76, 78, 79, 81, 82, 84, 86, 90–93, 96, 100], healthcare providers (13 studies, 17%) [30, 37, 39, 47, 59, 66, 67, 69, 71, 80, 87, 88, 95], or did not report (1 study, 1%) [77].

Twenty-one interventions included multiple target populations as participants. Common combinations of participants included parents and adolescents (11 studies) [43, 50–52, 61, 63, 75, 90–92, 100], adolescents and young adults (4 studies) [22, 26, 34, 73], clinicians and young adults (1 study) [47], parents and young adults (3 studies) [25, 78, 93], parents and clinicians (1 study) [66], and clinicians, adolescents, and young adults (1 study) [69]. Only three studies included only male adolescents or young adult study populations (2 were adolescents only, and the last one was both adolescents and young adults).

Eight of the 79 studies (10.1%) included a large proportion of parents from diverse racial and ethnic identities (defined as ≥ 50% other races than White) [33, 45, 56, 70, 76, 79, 81, 100], 6 (7.6%) included adolescents from diverse groups [27, 40, 64, 65, 74, 98], 8 (10.1%) included both parents and children from diverse groups [38, 51, 52, 61, 63, 75, 90, 91], 6 (7.6%) included young adults from diverse groups [26, 35, 57, 60, 80, 97], and 1 included both young adults and children from diverse groups (1.3%) [34].

### Socio-ecological levels

Based on a review of the reported intervention components, the audiences they targeted, and the socio-ecological model, most studies were conducted at the individual level (44, 55.7%), followed by interpersonal level (10, 12.7%), community level (3, 3.8%), and clinic level (4, 5.0%).

### Multi-level interventions

**Fig. 2:**
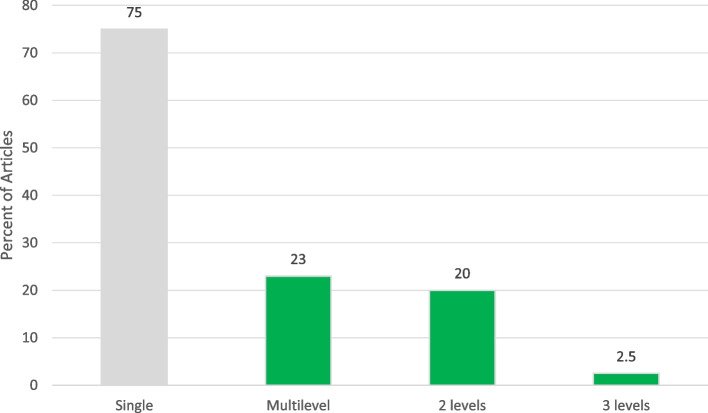
Levels of Interventions among Included Studies

Although most interventions were directed at a single level of the socio-ecologicl level (*n* = 61, 76.3%), 23.7% (*n*= 18) were multi-level. Sixteen (88.9%) combined two levels [27, 32, 39, 42, 43, 55, 63, 66, 69, 71, 73, 75, 77, 82, 98, 100], and 2 (9.1%) combined three levels (Fig. 2) [47, 67]. Common combinations of the levels included provider and clinical (5 studies) [66, 69, 71, 73, 82], interpersonal and clinical (4 studies) [27, 39, 43, 77], individual and interpersonal (2 studies) [32, 100], individual and clinical (2 studies) [42, 98], and individual and community (2 studies) [55, 75]. Meyer et. al aimed to use an electronic point-of-care prompt and 2-h lecture for providers to increase HPV vaccine uptake in retail clinics (provider and clinical interventions) [73]. Staras et al. sought to increase HPV vaccine initiation among publicly insured Florida adolescents ages 11–17 using a quasi-experimental factorial design with four study arms: 1) postcard campaign, 2) in-clinic Health Information Technology (HIT) system, 3) postcard campaign and in-clinic HIT system, and 4) usual care (individual and clinical interventions) [98]. Paskett et al. developed a program focused on HPV vaccine uptake among parents who have adolescent girls ages 9–17 who have not received the HPV vaccine, which would include vaccinations (individual and provider interventions) [82]. The 3-level combinations included: 1 study with individual, interpersonal, and clinical interventions [67], and 1 study with individual, clinical, and community interventions [47]. For example, Malo et al. created a 3-level intervention for parents to analyze which messages were most motivating to persuade them to administer the HPV vaccine to their child, for educating and training physicians, physician assistants, nurse practitioners and nurses who serve at primary clinics specialized in pediatrics or family medicine about the most persuasive messages in speaking to parents about the HPV vaccine for their children (individual, interpersonal, and clinical interventions) [67].

### Intervention components

The duration of interventions ranged from 10 min to 18 months among the studies reporting intervention time frames. Twenty-seven interventions (33.8%) reported using theory in intervention development [23, 24, 31, 35, 36, 40, 45, 47, 48, 50, 53, 54, 58, 61, 63, 67, 70, 72, 74–76, 81–83, 86, 100]. Theories or frameworks referenced included the Elaboration Likelihood Model, Culture-centric narrative theory, Health Belief Model, Theory of Reasoned Action/Planned Behavior, Moral Norm and Social Cognitive Theory.

Intervention components varied from education to offering vaccination (vaccine access). The most common intervention components were individual education of parents and/or adolescents (60, 76.0%); use of technology such as websites, PowerPoints, and text messages (21, 26.6%); and provider education (16, 20.3%). Examples of educational messaging were: expressing the benefit of the HPV vaccine, providing cervical and breast cancer prevention education, supplying educational handouts at an eighth-grade reading level, and displaying facts on posters about HPV and the HPV vaccine (i.e. both genders can receive the vaccine). The websites provided factual information on HPV and the HPV vaccine including statistics on the incidence of HPV infection and cervical cancer, risks associated with HPV infection, costs of vaccination, safety and efficacy of the HPV vaccine, and suggestions for how to talk to a doctor about the vaccine. Other components included patient reminders (13, 16.5%) [27, 50, 51, 62, 63, 70, 71, 74, 77, 83, 89, 90, 99], improving access to the HPV vaccine (6, 7.6%) [29, 55, 64, 75, 85, 89], health systems change (6, 7.6%) [43, 69, 75, 77, 81, 98], incentives (4, 5.1%) [46, 62, 68, 92], and community-wide campaigns or outreach (3, 3.8%) [32, 45, 75]. Patient reminders included phone calls, text messages, mailing reminders, and reminder-recall letters prompting adolescents to sign up for an appointment via a website. Several ways to improve access to the HPV vaccine consisted of utilizing school-based programs and expanding HPV vaccination programs in countries where there were no existing HPV vaccine programs. For incentives, gift cards (e.g., general merchandise and department stores, fashion and footwear retailers, bookstores, jewelry shops, motoring stores, and home improvement stores) and vaccine vouchers were used. Some studies combined two components (29, 36.7%) [24, 27–29, 31–35, 39, 40, 44, 45, 47, 50, 55, 65, 66, 71, 72, 81, 83, 85–88, 90, 97, 98], three components (6, 7.6%) [51, 62, 63, 69, 74, 82] or four components (3, 3.8%) [75, 77, 89]. Common intervention combinations included education and technology (18 studies, 23%) [24, 28, 31, 33–35, 40, 44, 51, 63, 65, 72, 74, 82, 86, 88, 89, 97], education and reminders (9 studies, 11%) [50, 51, 62, 63, 74, 77, 83, 89, 90], education and vaccine access (5 studies) [29, 55, 75, 85, 89], and provider education and technology (4 studies, 5%) [39, 69, 82, 87].

#### Community guide intervention categorization

**Fig. 3:**
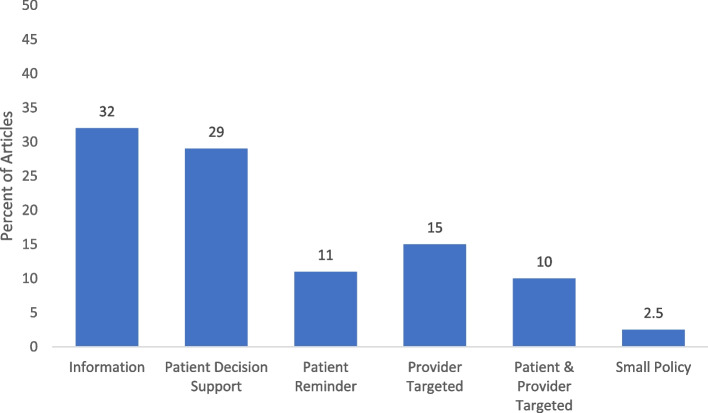
Intervention component categorizations based on community guide among included studies. Note. Interventions may have more than one intervention components

We reported on the categorization of the interventions based on the Community Guide’s categorization framework to assess the design and execution of health-related evidence-based interventions [12]. The most common type of HPV vaccination interventions were informational interventions (25, 31.7%). Of the behavioral interventions, 23 (29.1%) [24, 26, 32, 34–36, 38, 40, 44, 46, 48, 51, 52, 55, 61, 68, 76, 79, 81, 91, 92, 97, 100] were patient-targeted decision support, 9 (11.4%) [50, 62, 63, 70, 74, 83, 89, 90, 99] were patient-targeted reminders, 12 (15.2%) [22, 30, 37, 39, 43, 59, 67, 69, 71, 73, 87, 95] were provider-targeted, 8 (10.1%) [27, 47, 66, 75, 77, 82, 85, 98] were both patient and provider targeted interventions. Only 2 (2.5%) [29, 64] were related to environmental interventions related to small policies (i.e., organizational guidelines, no government involvement) (Fig. 3).

### Facilitators and barriers to intervention implementation

Several studies reported facilitators (13 studies, 16.5%) [26, 27, 29, 30, 32, 36, 37, 40, 45, 47, 48, 63, 65] and barriers (22 studies, 27.58%) [24, 26, 27, 30, 36, 37, 40, 43, 44, 50, 61, 67, 68, 73, 74, 77, 79, 87, 89, 93, 98, 99] to intervention implementation. Facilitators included use of patient navigators and user-friendly resources [26, 27, 36], interactive information sessions [29, 30, 37], low-cost interventions [30, 40, 47], and quality improvement initiatives [30, 37]. Barriers to implementation were related to cost [24, 27, 93, 99], time constraints with the given intervention [30, 36, 43, 67, 87] and integrating the intervention into clinical workflow [37, 73, 87, 98]. Other barriers included mobility of parents and technology challenges.

### HPV Intervention outcomes

Forty-two studies (53.2%) [22, 24, 26, 27, 29, 32, 34, 37–40, 44, 47, 48, 50–53, 55, 61–63, 68, 69, 71, 73–77, 79, 81–83, 85, 89–91, 97–100] reported on HPV vaccination outcomes, with 38 (48.1%) [22, 24, 26, 27, 29, 32, 34, 37–40, 44, 47, 50–53, 55, 61, 62, 68, 69, 71, 73–77, 79, 81–83, 85, 91, 97–100] reporting HPV vaccine initiation and 26 (32.9%) [22, 26, 27, 29, 34, 37–40, 48, 50, 52, 55, 63, 68, 71, 74–77, 81, 89–91, 97, 99, 100] reporting vaccine series completion. Post-intervention vaccine initiation ranged from 5% to 99.2%, while series completion ranged from 6.8% to 93%. For the experimental studies (*n*= 47), 11 (23.4%) measured vaccine initiation [24, 34, 38, 48, 51, 54, 61, 82, 83, 85, 86], and 3 (6.4%) measured completion [89, 90, 99]. Eleven (23.4%) assessed initiation and completion as outcomes (Table 2) [39, 40, 44, 50, 52, 53, 68, 71, 81, 91, 100]. Of the interventions that only measured vaccine initiation, 3 out of 11 (27%) found a significant increase in vaccine initiation [48, 82, 85]. For the interventions that measured both as an outcome, 3 out of the 11 (27%) found a significant increase in vaccine initiation [50, 71, 100]. Therefore, a total of 6 (12.8%) interventions demonstrated a significant increase in vaccine initiation [48, 50, 71, 82, 85, 100]. For the interventions that measured both vaccine initiation and completion, 1 (9.1%) reported a significant increase in completion only [81] and 2 (18.2%) in both vaccine initiation and completion [39, 68]. Of the interventions with quasi-experimental studies (*n* = 16), 5 (31.3%) were studies with comparison groups [30, 55, 62, 69, 98] and 11 (68.8%) were studies with pre and post intervention data collection (Table 3) [22, 25, 47, 59, 63, 73, 76, 79, 93, 95, 97]. Out of the quasi-experimental interventions with comparison groups (*n*= 5), 3 (60%) measured vaccine initiation [62, 69, 98], and 1 (20%) assessed both initiation and completion [55]. Of those, 2 (40%) demonstrated significant increase in vaccine initiation [62, 98], 0 in completion, and 1 (20%) in both as an outcome [55]. Out of the quasi-experimental interventions with pre and post-intervention designs (*n*= 11), 2 (18.2%) measured initiation [47, 79], 1 (9.1%) measured completion [63], and 4 (36.4%) assessed both as outcomes [22, 73, 76, 97]. One (9.1%) reported a significant increase in vaccine initiation [47] 1 (9.1%) in completion [63]; and 2 (18.2%) in both [73, 76].

**Table 2:** Significant HPV vaccine outcomes among experimental interventions

Study Design	Vaccine Outcomes	Significance	Multi Component	Multi Level
Experimental (*n* = 47)	11 (23.4%) measured Vaccine Initiation (VI) [26, 31, 44, 48, 52, 55, 58, 74, 79, 82, 100]	3 (27.0%) found a significant increase in VI [44, 74, 79]		
	3 (6.4%) measured Vaccine Completion (VC) [75, 78, 93]	None found a significant increase in VC		
	11 (23.4%) measured Vaccine Initiation and Vaccine Completion (both) [30, 35, 40, 46, 50, 51, 59, 64, 76, 85, 94]	3 (27.0%) found a significant increase in VI [46, 59, 94] 1 (9.1%) found a significant increase in VC [76] 2 (18.2%) found significant increases for both [30, 64]		
		**Total Significance:** 9 total articles had a significant increase in either VI, VC, or both [30, 44, 46, 59, 64, 74, 76, 79, 94]	6 (66.7%) were multi component [30, 46, 59, 74, 76, 79]	4 (44.4%) were multilevel [30, 59, 79, 94]

**Table 3:** Significant HPV vaccine outcomes among quasi-experimental interventions

Study Design	Quasi-experimental type	Vaccine Outcomes (VI, VC, Both)	Significance	Multi Component	Multi Level
Quasi-experimental (*n* = 16)	Comparison groups [53, 60, 65, 86, 92] **(** *n* **= **5; 31.3%)	3 (60.0%) measured Vaccine Initiation (VI) [60, 65, 92]	2 (40.0%) found a significant increase for VI [60, 92]		
		None for Vaccine Completion (VC)	^a^NA		
		1 (20.0%) measured Vaccine Initiation and Vaccine Completion (both) [53]	1 (20.0%) found both significantly increased [53]		
			**Total Significance:** 3 articles had a significant increase in either VI or both [53, 60, 92]	3 (100%) were multi component studies [53, 60, 92]	2 (66.7%) were multi-level [53, 92]
	Pre/Post Test [20, 21, 28, 37, 61, 66, 69, 70, 83, 87, 89] (*n* = 11; 68.8%)	2 (18.2%) measured Vaccine Initiation (VI) [28, 70]	1 (9.1%) found a significant increase for VI [28]		
		1 (9.1%) measured vaccine completion (VC) [61]	1 (9.1%) found a significant increase for VC [61]		
		4 (36.4%) measured both [20, 66, 69, 89]	2 (18.2%) found a significant increase for both [66, 69]		
			**Total Significance**: 4 total articles had significant increases in either VI, VC, or both [28, 61, 66, 69]	2 (50.0%) studies were multi component [28, 61]	3 (75.0%) were multi-level [28, 61, 69]

^a^ *NA* Not applicable

Other common intervention outcomes included measures of parental knowledge (18, 32.1%), self-efficacy (7, 12.5%), acceptability (7, 12.5%), and attitudes and beliefs (6, 10.7%). For adolescents, other outcome measures were knowledge (8, 34.5%), awareness (3, 13.0%), and attitudes and beliefs (3, 13.0%). For young adults, these measures included knowledge (14, 35.9%), attitudes and beliefs (7, 17.9%), and self-efficacy (4, 10.3%). Out of 79 studies, 15 (19%) measured vaccine intention.

### Quality assessment

The study quality (SQ) assessment included 12 criteria items with response options as 0 = no or 1 = yes. The results showed that SQ1 (the study had a clear objective) was the most common criterion met, with 79 (98.8%) studies meeting this criterion. This was followed by SQ3 (participants in the study are representative of those who would be eligible), which was met by 68 (85%) studies. SQ2 (eligibility criteria clearly described) and SQ6 (delivered consistently across the study population) were tied for third place, with 67 (83.75%) studies meeting these criteria. On the other hand, SQ8 (people assessing the outcomes blinded to participants' exposures/interventions) was the least met criterion, with only 9 (11.25%) studies meeting this criterion. SQ12 (the study took into account the use of individual-level data to determine effects at the group level) was met by 15 (18.75%) studies. SQ11 (outcome measured multiple times) was met by 19 (23.75%) studies, while SQ9 (loss to follow-up after baseline 20% or less) was met by 30 (37.50%) studies. Overall, 60% (*n* = 48) and 32.5% (*n* = 26)were rated as Good or Fair in quality, respectively. Six (0.75%) studies were rated as Poor. For a detailed presentation of the quality elements and overall quality scores, please refer to Supplementary Table 2.

## Discussion

We conducted a systematic review to assess interventions for HPV vaccine promotion. Our goal was to better describe common target populations of HPV vaccine interventions, common intervention levels and components, barriers and facilitators to intervention implementation, and the relationship between types of interventions and HPV-vaccine related outcomes. Previous systematic reviews have identified the breadth of intervention designs and contributed to our understanding of relative effectiveness of different intervention types [12, 14, 101]; however, given the advances in HPV vaccination research over the last several years, an update to these reviews was warranted. Moreover, previous systematic reviews have had a limited scope in terms of study settings, study designs, or topics and our goal here was to conduct a global and comprehensive review of interventions [14, 15, 102, 103]. We reported on the level of socio-ecological that each intervention targets, barriers and facilitators to the implementation of these interventions, and intervention with outcomes such as initiation and completion rates from the U.S. and other countries. In our update to these reviews, we found that while intervention components were described thoroughly to contribute to our knowledge of types of interventions being implemented, fewer details about barriers and facilitators and HPV vaccine-related outcomes (particularly vaccination rates) were included. There were few patterns to be discerned in which types of interventions were found to be most effective, and in fact, among those that did report, only 20.3% reported significant increases in either initiation or completion or both. Despite this, our findings offer six key insights into the types of interventions being implemented that make effective interventions.

From intervention research, we know that there are certain “components” that can help to promote successful intervention implementation and outcomes. For HPV vaccination specifically, we know that working with healthcare providers is an effective strategy [11]. More broadly, literature suggests that interventions are more effective when they focus on implementation at multiple levels [82] and use theory in intervention development [104]. However, in our review, we found that overall, many of the interventions identified did not adhere to these best practices; only 23% of the interventions were multi-level (18 total) and 34% employed theory (27 total).

We used the Community Guide and the Walling et al. systematic review classification of interventions such as informational, behavioral, and environmental to categorize and rank interventions [11]. Firstly, our review revealed the most commonly implemented interventions were not the types of interventions that had previously been shown to have the greatest impact. For example, while the success of behavioral provider and clinic-focused interventions (particularly ones that promote changes to systems like utilizing reminder-recall and encouraging strong recommendations) is well-documented [11], in our study we found other types of interventions were more often used. For example, information-providing interventions (used to increase knowledge of HPV, HPV-associated cancers and the HPV vaccine [11]) were most common (31.7%) followed by patient decision support interventions (29.1%). Among these intervention categories, the intensity of the activities ranged widely. For example, in our study among information-providing interventions some studies employed a passive approach by offering pamphlets and educational materials [60] whereas others were more active and included live presentations [57, 65]. Yet, educational, or information-giving interventions have been found to be less effective in increasing uptake or completion [103]. The interventions being implemented are not the types that have been shown to be most effective, which is consistent with other research that has identified a discrepancy between the implementation of interventions or strategies that are most effective compared to interventions that may be deemed “easiest” to implement [105, 106].

Secondly, despite extensive research showing the increased effectiveness of multi-level interventions [82], there were limited interventions included in this review that were multi-level (23%). For example, The Community Guide has found insufficient evidence for provider or patient education alone to increase vaccination, but it has found that using education in combination with provider-focused interventions (i.e. provider reminders; assessment and feedback) has been successful [107]. In this review, 75% of the interventions reported intervening on only a single level, most commonly in clinical or school-based settings focused on individuals or providers. Future interventions to promote HPV vaccination should prioritize intervening at multiple levels to more effectively improve vaccine outcomes and discern which combination of levels results in higher vaccination.

Thirdly, using theory is well-documented as a best practice in intervention development and implementation [104, 108]; however only one-third of the interventions in this review used theory in the design of their program strategies. It is highly possible that some of these interventions did in fact use theory or theoretical constructs to guide their research, but did not report it explicitly. The Health Belief Model, Theory of Planned Behavior, Social Cognitive Theory and the Elaboration Likelihood Model were the most commonly utilized; this is consistent with a recent systematic review exploring the use of theory in HPV vaccine interventions [102]. Using theory allows for understanding why specific interventions may be effective (or not effective) and for comparison across multiple studies. Thus, future HPV vaccine interventions should report more broadly on the use of theory in their intervention development and how constructs are employed in their design of intervention components or assessed in evaluation.

Fourthly, the effectiveness of these interventions was difficult to discern due to heterogeneity in measurement, outcomes, and study designs. Unfortunately, it is difficult to speak to what types of interventions were most effective as only about half reported on vaccine initiation (48%) and less than a third (32%) reported on vaccine series completion. Other commonly assessed outcomes included parental knowledge [33, 90, 91, 100], self-efficacy [35, 48, 54, 70, 72, 75, 76, 82, 86, 96], attitudes/beliefs [23, 31, 48, 49, 51, 54, 58, 65, 72, 75, 80, 82, 86, 97, 100], and acceptability [28, 34, 41, 43, 50, 72, 78, 79, 92]. There is mixed evidence on whether these outcomes are associated with uptake. For example, one meta-analysis found that parents’ beliefs, attitudes and intentions were positively associated with HPV vaccine uptake [109], while other studies have found intention to be unrelated to uptake, particularly in multivariable models, other factors seem to attenuate the effect of intention [110]. Moreover, many of the studies included in this review were quasi- or non-experimental, making it difficult to draw inferences about the effectiveness of any of the outcomes reported. Only about half focused on vaccine series initiation and completion. There are promising findings that a proportion of the interventions that reported significant changes in vaccination uptake or completion are multi-level and multi-component. Future intervention studies should focus on using rigorous methods to assess the effectiveness of different types of interventions, including investigating vaccination outcomes of series initiation or completion, and having longer-term follow-up to be able to assess longer-term outcomes. In addition, evaluation of multi-level interventions for the promotion of HPV vaccination should be conducted to contribute to their evidence of effectiveness.

Fifthly, related to the lack of reporting on intervention outcomes was a lack of reporting on implementation barriers and facilitators. Less than 20% of studies reported on facilitators and less than 30% reported on barriers. This is a similar finding to the review conducted by Smulian et al. (2016), who also reported a lack of reporting on barriers and facilitators [11]. This kind of information is critical in understanding program implementation, adaptation, and tailoring for different settings [24, 68, 93]. Recently, the use of hybrid trials, which can be used to assess both effectiveness and implementation outcomes, is emerging among implementation research [111, 112]. In the future, researchers could prioritize conducting these hybrid trials so that we can not only identify those interventions that are most effective, but also important implementation determinants that can inform sustainability and scalability in multiple types of healthcare settings.

Finally, it is important to note that it is a critical time, in the era of the COVID-19 pandemic to disseminate effective cancer prevention interventions. HPV vaccination rates have fallen during the pandemic [113, 114] and competing priorities have led to less time for clinics to devote to vaccine promotion [115]. Coupled with recent data suggesting that concerns about HPV vaccine safety are rising [116], this is indicative of a need to identify what works and how to implement it to prevent future generations from being susceptible to HPV-associated cancers. Overall, increased reporting of both vaccine outcomes as well as barriers and facilitators to vaccination will move the field forward and provide data to help researchers determine which types of interventions to prioritize.

### Strengths and limitations

Our study was strengthened by the inclusion of interventions globally and our focus on understanding multi-level intervention strategies. By categorizing interventions at different levels (e.g., individual, interpersonal, clinical) we have added to the growing literature on multi-level interventions. Additionally, almost 30% of the studies included were conducted outside of the United States. This finding helps to add to the growing global literature on HPV vaccine interventions and allows for comparability between the U.S. and other countries that continue to struggle with low HPV vaccination rates [2]. However, this should simultaneously be recognized as a potential limitation, as results may not be generalizable across all global geographies. While studies from North and South America, Europe, Africa, Asia, and Australia were included, there were only several from each continent (other than North America) which limits the generalizability of results. Similarly, less than 15% of studies included parents or children from diverse racial and ethnic identities (defined as ≥ 50% other races than White). This makes it hard to assess the impact of interventions for HPV vaccination on racially and ethnically diverse populations. Future HPV vaccination research should focus on these populations to test intervention effectiveness. We also were limited by only reporting on articles written in English and may be missing HPV vaccination interventions written in other languages.

Another key limitation is the lack of reporting vaccine-related outcomes in studies. Just over 50% reported either initiation and/or completion outcomes. This fact with varying study designs makes it difficult to collectively assess intervention effectiveness through data synthesis. Moreover, 40% of the studies were rated as “fair” or “poor” quality in our quality assessment, primarily due to studies not including multiple time points for outcome measures, not blinding participants in intervention studies, and for group-level studies not reporting on individual-level data to determine group-level effects. These limitations identify key gaps in the literature and that future research should focus on including more diverse populations in interventions, employing more rigorous study designs, and including vaccine initiation and completion rates.

## Conclusions

In 2020, the World Health Organization adopted a Global Strategy to eliminate cervical cancer, aiming for 90% of girls to be fully vaccinated by age 15 [2]. Given that males can suffer from HPV-associated cancers as well, many countries have expanded their vaccination programs to include males. However, worldwide, most countries fall far short of this 90% goal. Therefore, there is a strong need to expand implementation of HPV-vaccine promotion interventions beyond education alone and at a single level and use rigorous intervention designs. Inclusion of longer-term interventions and more evaluations focusing on vaccine initiation and/or completion would be helpful to truly understand what is most effective in improving HPV vaccination rates. Many of the interventions included in this review did not report vaccine uptake data; relied on strategies found to be less effective (e.g., education alone); did not use or not report on use of theory; did not report on barriers and facilitators to implementation; or addressed a single level for intervention. Improving on the design and evaluation of HPV vaccination interventions is particularly critical at this moment as many adolescents missed vaccinations during the COVID-19 pandemic and vaccine hesitancy is growing. Improving our understanding of which interventions to prioritize for implementation will be important to ensure future generations of adolescents are protected against HPV-associated cancers.

## Supplementary Information

**Additional files 1: Supplemental Table 1.** Systematic Review of HPV Vaccination Intervention Search Terms.** Supplemental Table 2.** Quality Assessment of Included Articles*.

## Data Availability

The datasets used and/or analyzed during the current study are available from the corresponding author on reasonable request.

## Abbreviations

COVID- 19: Coronavirus disease of 2019
HIT: Health Information Technology
HPV: Human papillomavirus
PRISMA: Preferred Reporting Items for Systematic Reviews and Meta-Analyses
