# Supplementary material for: A systematic review of interventions to promote HPV vaccination globally
Source: BMC Public Health. 2023 Jun 29;23:1262. doi: 10.1186/s12889-023-15876-5 (PMC10308645; doi:10.1186/s12889-023-15876-5)
Supplement: Supplementary file 1 — Additional files 1: Supplemental Table 1. Systematic Review of HPV Vaccination Intervention Search Terms. Supplemental Table 2. Quality Assessment of Included Articles*. [file 12889_2023_15876_MOESM1_ESM.docx]

Supplemental Table 1. Systematic Review of HPV Vaccination Intervention Search Terms

| Bibliographic Database and Keywords |
| --- |
| **Medline/PubMed**  (child[tw] OR children[tw] OR childhood[tw] OR pediatric[tw] OR paediatric[tw] OR adolescent*[tw] OR adolescence[tw] OR teenager*[tw] OR teen* [tw] OR youth[tw] OR young adult[tw] OR school age*[tw] OR juvenile*[tw] OR parent*[tw] OR family[tw] OR families[tw] OR student*[tw]) AND (public health[tw] OR intervention*[tw] OR school*[tw]OR education[tw] OR community*[tw] OR prevent[tw] OR preventing[tw] OR prevention[tw] OR program*[tw] OR train[tw] OR training[tw] OR behaviour*[tw] OR behavior*[tw] OR behavioral therapy[tw] OR social environment*[tw] OR informational[tw] OR Reminder*[tw] OR message*[tw] OR text messaging[tw] OR electronic OR messaging[tw] OR health education[tw] OR consumer education[tw] OR delivery[tw] OR communication*[tw] OR strategy[tw] OR promotion[tw] OR promoting[tw]) AND (Human papilloma virus[tw] OR papillomaviridae[tw] OR papilloma[tw] OR HPV[tw] OR Papillomavirus[tw]) AND (Papillomavirus Vaccines[tw] OR vaccinate*[tw] OR vaccines[tw] OR vaccination[tw] OR immunize[tw] OR immunized[tw] OR immunization[tw] OR immunise[tw] OR immunised[tw] OR immunisation[tw] OR prophylaxis[tw]) AND (Uptake[tw] OR coverage[tw] OR compliance[tw] OR completion[tw]) AND (awareness[tw] OR comprehension[tw] OR understanding[tw] OR acceptance[tw] OR perception[tw] OR knowledge[tw]) NOT ("Animals"[Mesh] NOT ("Animals"[Mesh] AND "Humans"[Mesh])) |
| **CINAHL**  (child OR children OR childhood OR pediatric OR paediatric OR adolescent* OR adolescence OR teenager* OR teen* OR youth OR young adult OR school age* OR juvenile* OR parent* OR family OR families OR student*) AND (public health OR intervention* OR school*OR education OR community* OR prevent OR preventing OR prevention OR program* OR train OR training OR behaviour* OR behavior* OR behavioral therapy OR social environment* OR informational OR Reminder* OR message* OR text messaging OR electronic OR messaging OR health education OR consumer education OR delivery OR communication* OR strategy OR promotion OR promoting) AND (Human papilloma virus OR papillomaviridae OR papilloma OR HPV OR Papillomavirus) AND (Papillomavirus Vaccine* OR vaccinate OR vaccinated OR vaccination OR immunize OR immunized OR immunization OR immunise OR immunised OR immunisation OR prophylaxis) AND (Uptake OR coverage OR compliance OR completion) AND (awareness OR comprehension OR understanding OR acceptance OR perception OR knowledge) NOT ("Animals" NOT ("Animals" AND "Humans")) |
| **EMBASE**  (('child'/exp OR child OR 'children'/exp OR children OR 'childhood'/exp OR childhood OR 'pediatric'/exp OR pediatric OR 'paediatric'/exp OR paediatric OR adolescent* OR 'adolescence'/exp OR adolescence OR teenager* OR teen* OR 'youth'/exp OR youth OR young) AND ('adult'/exp OR adult) OR 'school'/exp OR school) AND age* OR juvenile* OR parent* OR 'family'/exp OR family OR 'families'/exp OR families OR student*  AND  ((((((('public'/exp OR public) AND ('health'/exp OR health) OR intervention* OR school*or) AND ('education'/exp OR education) OR community* OR prevent OR preventing OR 'prevention'/exp OR prevention OR program* OR 'train'/exp OR train OR 'training'/exp OR training OR behaviour* OR behavior* OR behavioral) AND ('therapy'/exp OR therapy) OR 'social'/exp OR social) AND environment* OR informational OR reminder* OR message* OR text) AND messaging OR electronic OR messaging OR 'health'/exp OR health) AND ('education'/exp OR education) OR 'consumer'/exp OR consumer) AND ('education'/exp OR education) OR 'delivery'/exp OR delivery OR communication* OR 'strategy'/exp OR strategy OR 'promotion'/exp OR promotion OR promoting  AND  ('papillomavirus'/exp OR papillomavirus) AND ('vaccines'/exp OR vaccines) OR vaccinate OR vaccinated OR 'vaccination'/exp OR vaccination OR immunize OR immunized OR 'immunization'/exp OR immunization OR immunise OR immunised OR 'immunisation'/exp OR immunisation OR 'prophylaxis'/exp OR prophylaxis  AND  uptake OR coverage OR 'compliance'/exp OR compliance OR 'completion'/exp OR completion  AND  'awareness'/exp OR awareness OR 'comprehension'/exp OR comprehension OR 'understanding'/exp OR understanding OR 'acceptance'/exp OR acceptance OR 'perception'/exp OR perception OR 'knowledge'/exp OR knowledge  AND  #1 AND #2 AND #3 AND #4 AND #5 AND #6  AND  #7 AND 'human'/de  AND  #8 AND (2015:py OR 2016:py OR 2017:py OR 2018:py OR 2019:py OR 2020:py) |
| **Web of Science TOPIC:** ((child  OR children  OR childhood  OR pediatric  OR paediatric  OR adolescent*  OR adolescence  OR teenager*  OR teen*  OR youth  OR young adult  OR school age*  OR juvenile*  OR parent*  OR family  OR families  OR student*)  AND (public health  OR intervention*  OR school*OR education  OR community*  OR prevent  OR preventing  OR prevention  OR program*  OR train  OR training  OR behaviour*  OR behavior*  OR behavioral therapy  OR social environment*  OR informational  OR Reminder*  OR message*  OR text messaging  OR electronic  OR messaging  OR health education  OR consumer education  OR delivery  OR communication*  OR strategy  OR promotion  OR promoting)  AND (Human papilloma virus  OR papillomaviridae  OR papilloma  OR HPV  OR Papillomavirus)  AND (Papillomavirus Vaccine*  OR vaccinate  OR vaccinated  OR vaccination  OR immunize  OR immunized  OR immunization  OR immunise  OR immunised  OR immunisation  OR prophylaxis)  AND (Uptake  OR coverage  OR compliance  OR completion)  AND (awareness  OR comprehension  OR understanding  OR acceptance  OR perception  OR knowledge)  NOT ("Animals"  NOT ("Animals"  AND "Humans")))  **Cochrane Reviews and Cochrane Trials**  ((child OR children OR childhood OR pediatric OR paediatric OR adolescent* OR adolescence OR teenager* OR teen* OR youth OR young adult OR school age* OR juvenile* OR parent* OR family OR families OR student*) AND (public health OR intervention* OR school*OR education OR community* OR prevent OR preventing OR prevention OR program* OR train OR training OR behaviour* OR behavior* OR behavioral therapy OR social environment* OR informational OR Reminder* OR message* OR text messaging OR electronic OR messaging OR health education OR consumer education OR delivery OR communication* OR strategy OR promotion OR promoting) AND (Human papilloma virus OR papillomaviridae OR papilloma OR HPV OR Papillomavirus) AND (Papillomavirus Vaccine* OR vaccinate OR vaccinated OR vaccination OR immunize OR immunized OR immunization OR immunise OR immunised OR immunisation OR prophylaxis) AND (Uptake OR coverage OR compliance OR completion) AND (awareness OR comprehension OR understanding OR acceptance OR perception OR knowledge) NOT ("Animals" NOT ("Animals" AND "Humans"))) in Title Abstract Keyword - (Word variations have been searched) |
| **SCOPUS**  ( ( uptake OR coverage OR compliance OR completion ) ) AND ( ( awareness OR comprehension OR understanding OR acceptance OR perception OR knowledge ) ) AND ( ( "public health" OR intervention OR school OR education OR community OR prevent OR preventing OR prevention OR program OR train OR training OR behaviour OR behavior OR "behavioral therapy" OR "social environment" OR informational OR reminder OR message OR "text messaging" OR "text messages" OR electronic OR "mobile device" OR "mobile application" OR "cellular device" OR cellular AND messaging OR "cell phone" OR "mobile phone" OR "social media" OR messaging OR "health education" OR consumer AND education OR delivery OR communication OR strategy OR promotion OR promoting OR motivational ) ) AND ( ( "Human papilloma virus" OR papillomaviridae OR papilloma OR hpv OR papillomavirus ) ) AND ( ( "Papillomavirus Vaccines" OR vaccinate OR vaccinated OR vaccination OR immunize OR immunized OR immunization OR immunise OR immunised OR immunisation OR prophylaxis ) ) AND ( ( child OR children OR childhood OR pediatric OR paediatric OR adolescent OR adolescence OR teenager OR teen OR youth OR "young adult" OR school AND age OR juvenile OR parent OR family OR families OR student ) ) AND ( LIMIT-TO ( PUBYEAR , 2020 ) OR LIMIT-TO ( PUBYEAR , 2019 ) OR LIMIT-TO ( PUBYEAR , 2018 ) OR LIMIT-TO ( PUBYEAR , 2017 ) OR LIMIT-TO ( PUBYEAR , 2016 ) OR LIMIT-TO ( PUBYEAR , 2015 ) ) AND ( LIMIT-TO ( LANGUAGE , "English" ) ) AND ( LIMIT-TO ( SUBJAREA , "AGRI" ) OR LIMIT-TO ( SUBJAREA , "BIOC" ) OR LIMIT-TO ( SUBJAREA , "COMP" ) OR LIMIT-TO ( SUBJAREA , "DECI" ) ) |

Supplemental Table 2. Quality Assessment of Included Articles*

| **First Author, Year** | **SQ1: Clear Obj** | **SQ2: Eligibility** | **SQ3: Represent** | **SQ4: Enrollment** | **SQ5: Sample Size** | **SQ6: Delivery** | **SQ7: Outcome Measures Assessed** | **SQ8: Blinded** | **SQ9: LTFU** | **SQ10: Stat Method** | **SQ11: Measured Multiple Times** | **SQ12: Ind/Group Data** | **Quality Rating** |
| --- | --- | --- | --- | --- | --- | --- | --- | --- | --- | --- | --- | --- | --- |
| Austin, 2019 | CD^3^ | 0 | 1 | 0 | 1 | NR^1^ | 1 | 0 | 1 | 1 | 0 | NA^2^ | Poor |
| Baxter, 2011 | 1 | 1 | 1 | 1 | 1 | 1 | 1 | 0 | NR | 1 | 0 | NA | Fair |
| Bennett, 2015 | 1 | 1 | 1 | 1 | 1 | 1 | 1 | 0 | 0 | 1 | 1 | NA | Good |
| Berenson, 2015 | 1 | 1 | 1 | NR | 1 | 1 | 1 | 0 | 1 | 1 | 0 | NA | Fair |
| Berenson, 2016 | 1 | 1 | 1 | 1 | 1 | 1 | 1 | 0 | 1 | 1 | 0 | NA | Good |
| Berenson, 2019 | 1 | 1 | 1 | 1 | 1 | 1 | 1 | 0 | 1 | 1 | 0 | 0 | Good |
| Bonafide, 2015 | 1 | 1 | 0 | 1 | 1 | 1 | 1 | 0 | NR | 1 | 0 | NA | Good |
| Botha, 2015 | 1 | 1 | 0 | 1 | 1 | 1 | NA | NA | NR | 0 | 0 | NA | Fair |
| Calo, 2018 | 1 | 1 | 1 | 1 | 1 | 1 | 1 | 0 | NR | 1 | NA | 1 | Good |
| Carolan, 2018 | 1 | 1 | 1 | 1 | 0 | 1 | 1 | 1 | 1 | 1 | 1 | NA | Good |
| Chigbu, 2017 | 1 | 1 | 1 | 1 | 1 | 1 | 1 | 0 | NA | 1 | 1 | 1 | Good |
| Cipriano, 2018 | 1 | 1 | 1 | 1 | CD | 1 | 1 | 0 | NA | 1 | 0 | NA | Fair |
| Cory, 2019 | 1 | 1 | 1 | 1 | 1 | 1 | 1 | NR | NA | 1 | 0 | NA | Good |
| Darville, 2018 | 1 | 1 | 1 | 1 | 0 | 1 | 0 | 0 | NR | NR | 1 | 0 | Fair |
| Davies, 2017 | 1 | 1 | 1 | 1 | 1 | 1 | 1 | NR | NR | 1 | 0 | 1 | Good |
| Dawson, 2017 | 1 | 1 | 1 | 1 | 1 | 1 | 1 | NA | 0 | 1 | 1 | 1 | Good |
| Dempsey, 2019 | 1 | 1 | 1 | 1 | 1 | 1 | 1 | 1 | 1 | 1 | 1 | CD | Good |
| Dempsey, 2018 | 1 | 1 | 1 | 1 | 1 | 1 | 1 | CD | CD | 1 | 0 | 1 | Good |
| DiClemente, 2015 | 1 | 1 | 1 | 1 | 1 | 1 | 1 | 0 | 1 | NA | NA | 1 | Good |
| Donahue, 2018 | 1 | 1 | 1 | 1 | NR | 1 | 1 | NR | 1 | 1 | 0 | CD | Good |
| Dreyer, 2015 | 1 | 1 | 1 | CD | 1 | 1 | 1 | 0 | NA | 1 | 0 | NA | Good |
| Edwards, 2019 | 1 | 1 | 1 | 1 | NR | 1 | 1 | CD | 1 | 0 | CD | NA | Fair |
| Esposito, 2018 | 1 | 1 | 1 | 1 | 1 | 1 | 1 | 1 | 1 | 0 | 1 | CD | Good |
| Ford, 2020 | 1 | 1 | 1 | 1 | 1 | 1 | 1 | NA | 1 | NR | 0 | NA | Good |
| Forster, 2017 | 1 | 1 | 1 | 1 | 0 | 1 | 1 | 0 | 1 | 0 | 0 | 0 | Fair |
| Gerent, 2020 | 1 | CD | 1 | CD | CD | CD | 0 | NA | NA | 0 | NA | CD | Poor |
| Grandahl, 2016 | 1 | 1 | 1 | 1 | 1 | 1 | 0 | NA | 1 | 1 | 0 | 1 | Good |
| Gualano, 2019 | 1 | 1 | 1 | 1 | CD | 1 | 0 | NA | CD | 1 | 0 | 1 | Fair |
| Henrikson, 2018 | 1 | 1 | 1 | 1 | 0 | 1 | 1 | NA | 0 | 1 | NA | NA | Good |
| Hofstetter, 2017 | 1 | 1 | 1 | 0 | 1 | 1 | 0 | 0 | 1 | 1 | 0 | NA | Fair |
| Juraskova, 2011 | 1 | 1 | 0 | 1 | 1 | 1 | 1 | CD | 0 | 1 | 0 | NA | Fair |
| Juraskova, 2012 | 1 | 0 | 0 | 1 | 0 | 1 | 1 | 1 | 0 | 1 | 0 | NA | Fair |
| Kaul, 2019 | 1 | NA | 1 | NA | 1 | 1 | 1 | NA | NA | 1 | 1 | CD | Good |
| Kepka, 2011 | 1 | 1 | 0 | 1 | 0 | 1 | 1 | NR | 0 | 1 | 0 | NA | Good |
| Kester, 2013 | 1 | 1 | 1 | 1 | 1 | 1 | 1 | NR | NA | 1 | 0 | NA | Good |
| Kim, 2016 | 1 | 1 | 0 | 1 | 0 | 1 | 1 | NA | NA | NA | NA | NA | Good |
| Kumar, 2019 | 1 | 0 | 1 | CD | 1 | 1 | 1 | NA | 0 | 1 | 0 | 0 | Good |
| Kwang, 2016 | 1 | 1 | 1 | 1 | NR | 1 | 1 | 0 | 1 | 1 | 0 | NA | Good |
| Lee, 2018 | 1 | 1 | 1 | 1 | 1 | 1 | CD | 0 | 0 | 0 | 1 | NA | Fair |
| Lefevere, 2015 | 1 | 1 | 1 | 1 | 1 | 1 | 1 | 0 | NA | 1 | 1 | 0 | Good |
| Lennon, 2019 | 1 | 1 | 1 | 1 | 1 | CD | 1 | 0 | NR | 1 | 1 | CD | Good |
| Lin, 2019 | 1 | CD | CD | CD | CD | 1 | 1 | 0 | NA | CD | 1 | NA | Fair |
| Liu, 2019 | 1 | 1 | 1 | 1 | 1 | 1 | 1 | 1 | 1 | 1 | NR | NA | Good |
| Malo, 2016 | 1 | 1 | 1 | 1 | 1 | 1 | 1 | NA | NA | NA | 0 | CD | Fair |
| Malo, 2018 | 1 | 1 | 1 | 1 | 1 | 1 | 1 | 1 | 1 | 1 | 1 | 1 | Good |
| Mantzari, 2014 | 1 | 1 | 1 | 0 | 1 | 1 | 1 | 1 | NR | 1 | 0 | NR | Fair |
| Marchand- Ciriello, 2019 | 1 | CD | 1 | CD | 0 | CD | 0 | 0 | NA | 1 | NA | 1 | Fair |
| McGlone, 2017 | 1 | 1 | 1 | 1 | 1 | 1 | 1 | NR | NR | CD | 0 | NA | Good |
| McLean, 2017 | 1 | 1 | 1 | 1 | 1 | 1 | 1 | NR | 1 | 1 | 1 | 1 | Good |
| McRee, 2018 | 1 | 1 | 1 | 1 | 1 | 1 | 1 | NR | 1 | 1 | 0 | NA | Good |
| Meyer, 2018 | 1 | 1 | 1 | 1 | 1 | 1 | 1 | NR | 1 | 1 | 1 | 1 | Good |
| Mohanty, 2018 | 1 | 1 | CD | CD | CD | CD | CD | NR | CD | CD | 0 | 0 | Fair |
| Molokwu, 2019 | 1 | 1 | 1 | 1 | CD | CD | CD | CD | CD | 1 | 0 | NA | Poor |
| Morales- Campos, 2017 | 1 | 1 | 1 | CD | CD | 1 | 1 | NR | 0 | 1 | 0 | NA | Fair |
| Nissen, 2019 | 1 | 1 | 1 | 1 | 1 | CD | CD | NR | 1 | 0 | 1 | 1 | Good |
| Nwanodi, 2017 | 1 | 1 | 1 | 1 | 1 | CD | CD | CD | 0 | 1 | 0 | 0 | Fair |
| Obulaney, 2016 | 1 | 0 | CD | CD | CD | CD | 1 | NA | 1 | 1 | 0 | NA | Poor |
| Padmanabha, 2019 | 1 | 1 | 0 | 1 | 1 | 1 | 1 | NA | NR | 1 | 0 | NA | Fair |
| Parra- Medina, 2015 | 1 | 1 | 1 | 1 | 1 | 1 | 0 | 0 | 0 | NA | NA | NA | Good |
| Paskett, 2016 | 1 | 1 | 1 | 1 | 1 | NR | 1 | NR | 1 | 1 | 0 | NA | Good |
| Patel, 2012 | 1 | 1 | 1 | 1 | 1 | 1 | CD | NR | NR | 1 | 0 | NA | Good |
| Pierre- Joseph, 2016 | 1 | 1 | 1 | 1 | 1 | 1 | 1 | 0 | 1 | 1 | 0 | NA | Good |
| Porter, 2018 | 1 | 1 | 1 | 1 | 1 | 1 | 1 | NR | 1 | 1 | NA | NA | Good |
| Poscia, 2019 | 1 | 1 | 1 | 1 | 0 | CD | 1 | 0 | NR | 1 | 0 | NA | Fair |
| Pot, 2017 | 1 | 1 | 1 | 1 | 1 | 1 | 1 | NR | 1 | 1 | 0 | NA | Good |
| Reno, 2018 | 1 | 1 | 1 | 1 | NR | 1 | CD | NR | NR | 1 | 1 | 1 | Fair |
| Rhodes, 2017 | 1 | 0 | 1 | CD | 1 | 1 | 1 | NA | 0 | 1 | 0 | NA | Fair |
| Richman, 2016 | 1 | 1 | 1 | 1 | 1 | 1 | 1 | 1 | 0 | 1 | 0 | NA | Good |
| Richman, 2019 | 1 | 1 | 1 | NR | 1 | 1 | 1 | 1 | 0 | 1 | 1 | NA | Good |
| Rickert, 2015 | 1 | 1 | 1 | 1 | 1 | 1 | 1 | 0 | 0 | CD | 0 | NA | Good |
| Rockliffe, 2018 | 1 | 0 | 1 | CD | 1 | 1 | 1 | 0 | CD | 0 | 0 | 0 | Good |
| Roussos- Ross, 2017 | 1 | 1 | 1 | 1 | 1 | 1 | 1 | 0 | NR | 1 | 0 | 0 | Good |
| Sadoh, 2018 | 1 | 0 | 1 | CD | 1 | 1 | 1 | 0 | NR | 1 | 0 | NA | Fair |
| Schnaith, 2018 | 1 | 1 | CD | NR | NA | CD | 1 | NA | NA | 1 | 0 | NA | Fair |
| Shah, 2019 | 1 | CD | 1 | 1 | CD | 1 | CD | NR | 1 | 1 | 0 | NA | Fair |
| Staples, 2018 | 1 | 0 | CD | CD | CD | CD | 0 | 0 | 0 | 0 | 0 | NA | Poor |
| Staras, 2013 | 1 | 1 | 1 | 1 | 1 | 1 | 1 | 0 | 1 | 1 | 0 | 1 | Good |
| Stern, 2014 | 1 | 1 | 1 | 1 | 1 | 1 | 1 | 0 | 1 | 1 | 0 | 0 | Good |
| Underwood, 2015 | 1 | 1 | 1 | 1 | 1 | 1 | 1 | 0 | NA | 1 | 1 | NA | Good |
| **Totals:** | | | | | | | | | | | | | |
| Good | - | - | - | - | - | - | - | - | - | - | - | - | 48 |
| Fair | - | - | - | - | - | - | - | - | - | - | - | - | 26 |
| Poor | - | - | - | - | - | - | - | - | - | - | - | - | 5 |

Note. * These metrics were established from the SQ1-10 scores. ^1^ NR= Not Reported. ^2^ NA = Not Applicable.^3^ CD = Can’t Determine.
